# Supplementary material for: Minimizing interfacial energy losses via multifunctional cage-like diammonium molecules for efficient perovskite/silicon tandem solar cells
Source: Nat Commun. 2025 Sep 30;16:8692. doi: 10.1038/s41467-025-63720-8 (PMC12484657; doi:10.1038/s41467-025-63720-8)
Supplement: Supplementary file 1 — Supplementary Information [file 41467_2025_63720_MOESM1_ESM.pdf]

Supplementary Information for

Minimizing Interfacial Energy Losses via Multifunctional Cage-like Diammonium Molecules for Efficient Perovskite/Silicon Tandem Solar Cells

Xin Li[1,3], Zhiqin Ying[1,*], Linhui Liu[1], Jun Wu[1], Haofan Ma[1], Ziyu He[1], Yunyun Yu[1], Yihan Sun[1], Meili Zhang[1], Xuchao Guo[1], Yuheng Zeng[1], Xi Yang[1,2,*], Jichun Ye[1,2,*]

[1]Zhejiang Provincial Engineering Research Center of Energy Optoelectronic Materials and Devices, Ningbo Institute of Materials Technology and Engineering, Chinese Academy of Sciences, Ningbo 315201, China.

[2]Research Center for Wide Bandgap Semiconductors and Devices, YongJiang Laboratory, Ningbo 315201, Zhejiang, China

[3]University of Chinese Academy of Sciences, No.19(A) Yuquan Road, Shijingshan District, Beijing 100049, China

*Corresponding author:
E-mail: yingzhiqin@nimte.ac.cn; yangx@nimte.ac.cn; jichun.ye@nimte.ac.cn

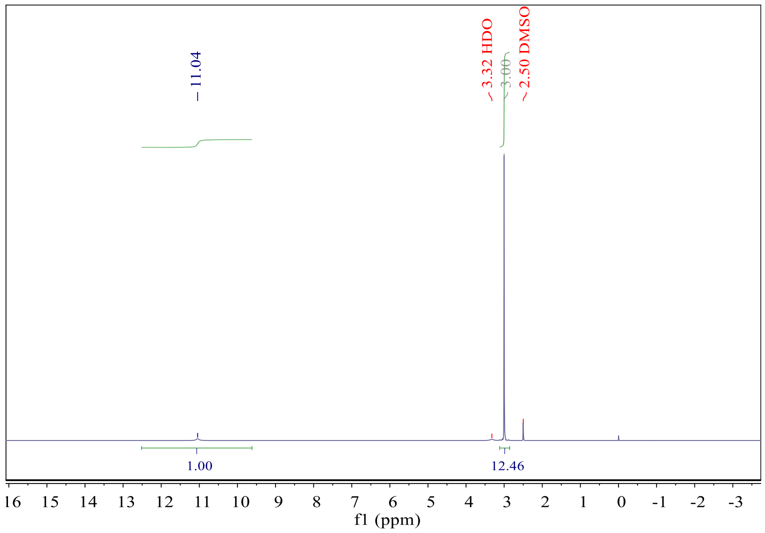

Supplementary Fig. 1 ^{1}H NMR characterisation of DCl after synthesis.

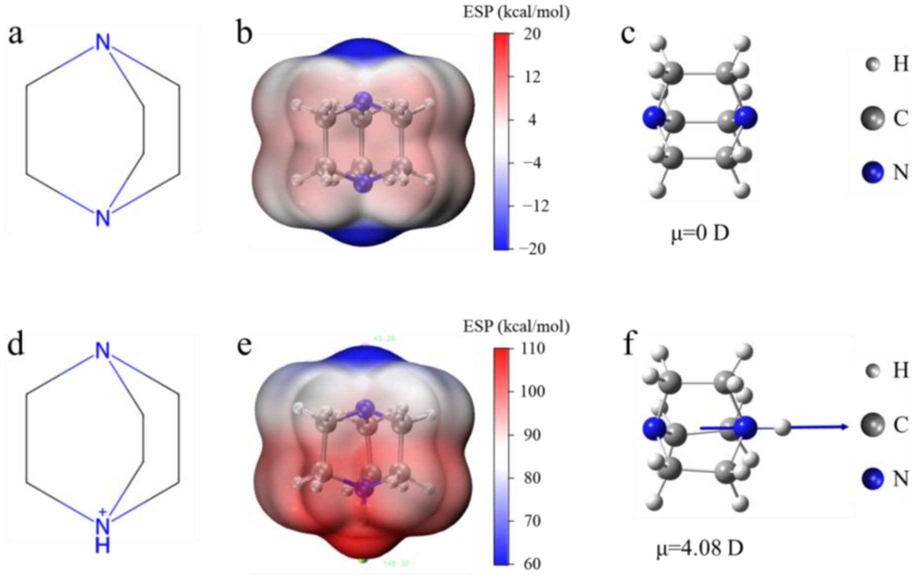

Supplementary Fig. 2 (a, d) Chemical structures, (b, e) electrostatic potentials (ESPs) and (c, f) corresponding electric dipole moments of both (a-c) 1,4-diazabicyclo[2.2.2]octane and (d-f) cage-like diammonium cation. Light gray, dark gray and blue balls represent the H, C, and N atoms, respectively.

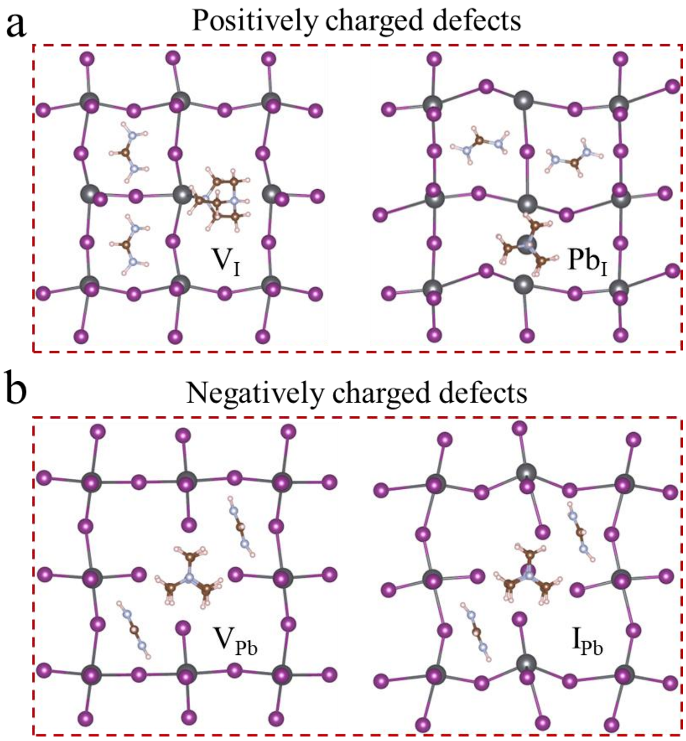

Supplementary Fig. 3 Top-view of various types of defects passivated by DCl. (a) Positively charged defects. (b) Negatively charged defects. Pink, light brown, light gray, violet, and gray balls represent the H, C, N, I and Pb atoms, respectively.

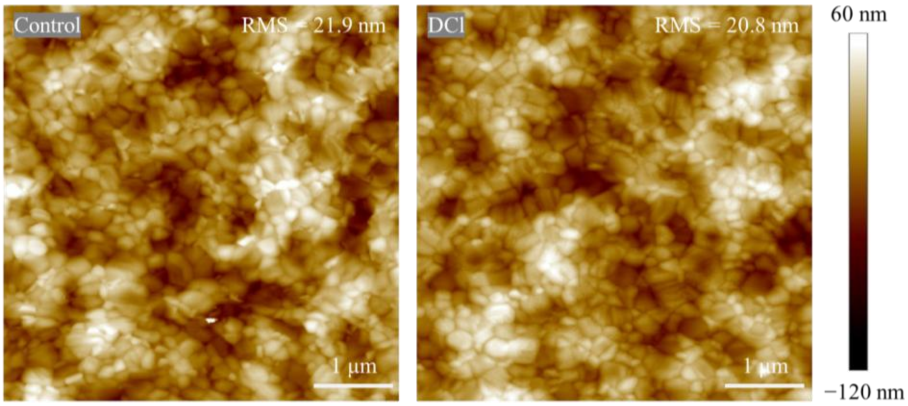

Supplementary Fig. 4 AFM images of control and DCl-treated perovskite films with a scan area of 5 μm × 5 μm.

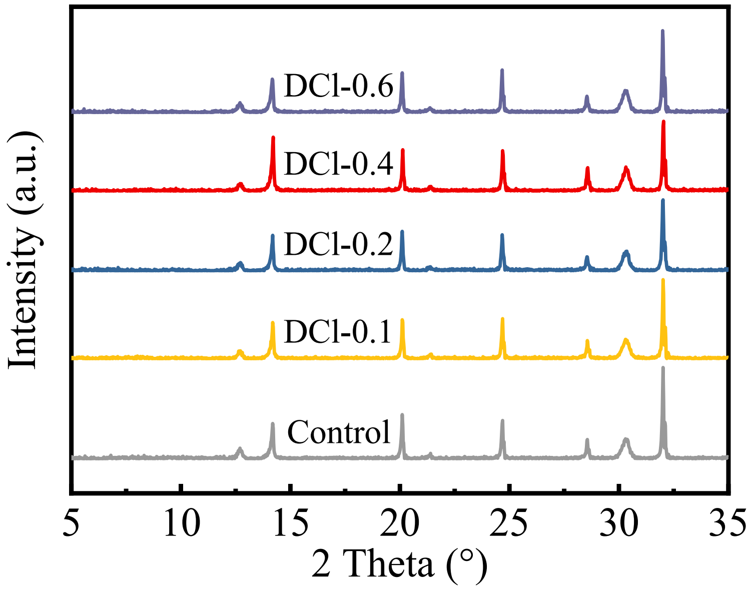

Supplementary Fig. 5 XRD patterns of the control and DCl-treated perovskite films.

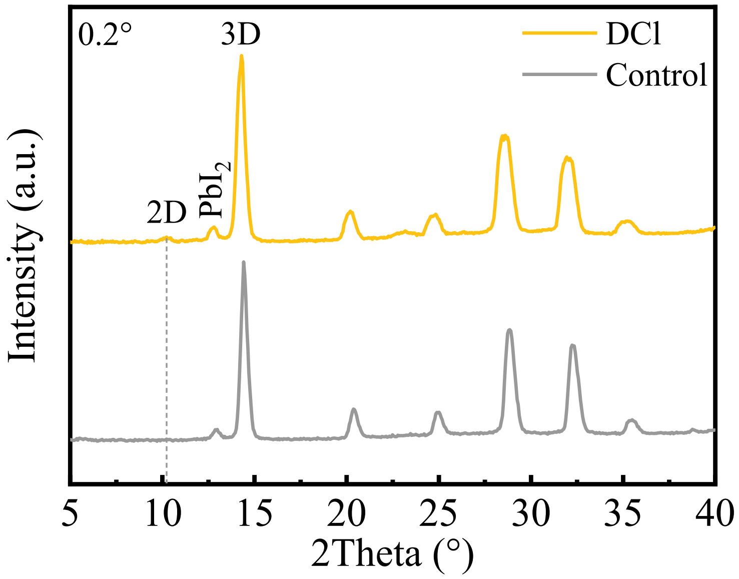

Supplementary Fig. 6 GIXRD patterns of the control and DCl-treated perovskite films with a grazing incidence angle of 0.2°.

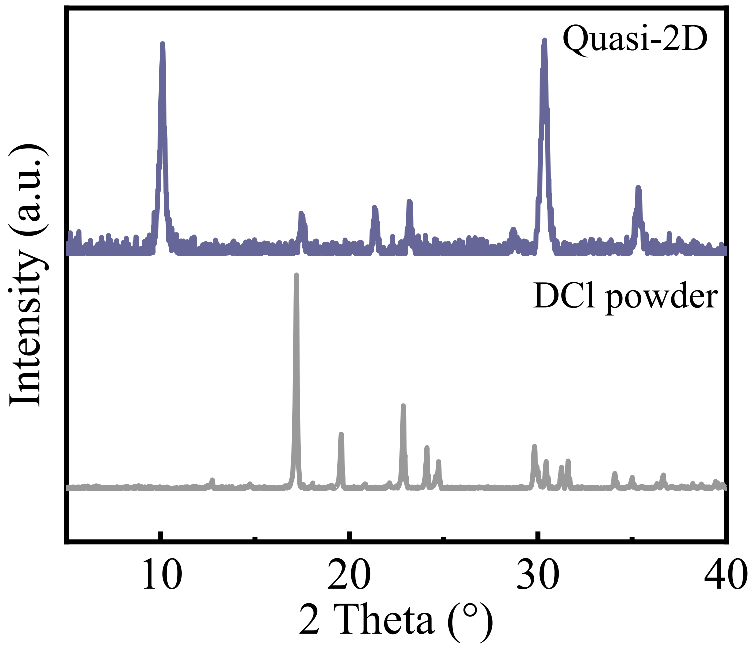

Supplementary Fig. 7 XRD patterns of DCl powder and synthesized single-crystal 2D perovskite. Notably, small grains on the DCl-treated perovskite surface (Fig. 1a and b) should be ascribed to the formation of a 2D phase rather than the post-treated DCl material itself, as evidenced in Supplementary Fig. 7. The peak position at 10.2° of the synthesized single-crystal 2D perovskite precisely matches that observed in the GIXRD measurement (Supplementary Fig. 6).

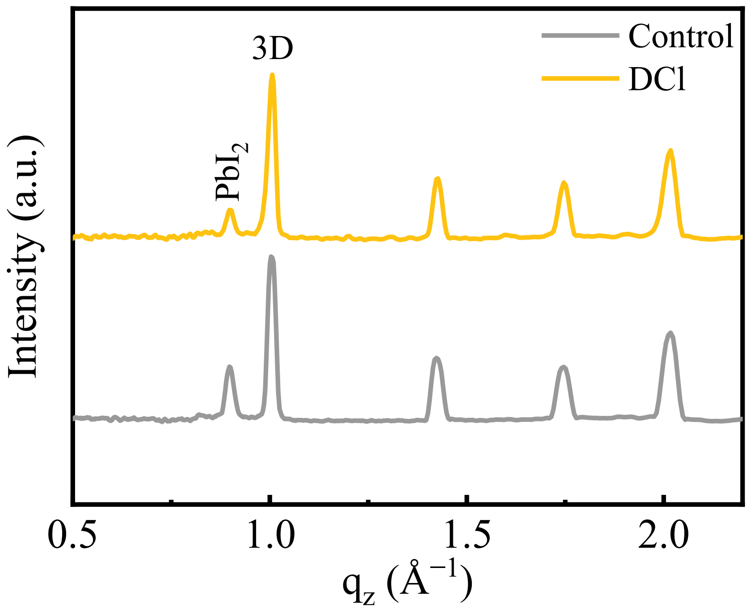

Supplementary Fig. 8 The (001) out-of-plane line cuts of GIWAXS images of the control and DCl-treated perovskite films.

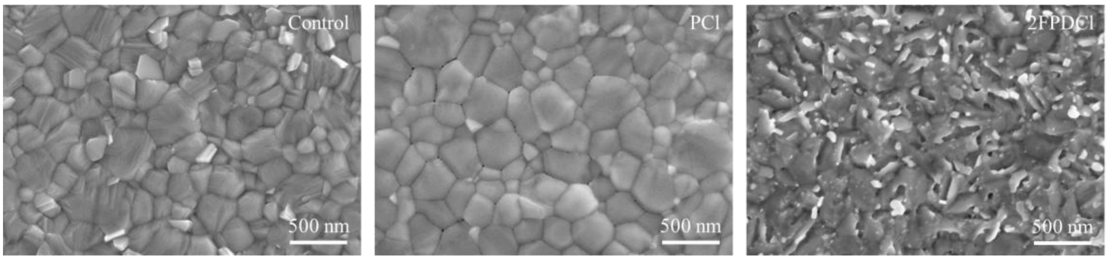

Supplementary Fig. 9 SEM images of the control, PCl-treated and 2FPDCl-treated perovskite films. To highlight the uniqueness of our designed DCl, we additionally selected other two similar molecules-4,4-difluoropiperidine hydrochloride (2FPDCl)[1,2] and piperazinium hydrochloride (PCl)[3] containing the same Cl anion as DCl for comparison. Similar to DCl, both 2FPDCl and PCl incorporate Lewis acid and Lewis base functional groups within their cationic moieties. For the control sample, the perovskite surface consists of clear steps (or terraced edges) and discontinuous PbI_2 filling the grain boundaries. In contrast, the PCl treatment drastically smooths out these steps and reacts with residual PbI_2, which is in line with our previous report[3]. For the 2FPDCl-treated perovskite film, platelet-like substances with significantly different sizes are observed on the surface compared to the control and PCl-treated samples, which is due to the formation of $(2FPD)_2PbI_4$ 2D phase on the surface of the 2FPDCl-treated sample[1,2]. Obviously, both PCl-treated and 2FPDCl-treated samples show different surface morphologies in comparison with the DCl-treated sample (Fig. 1a).

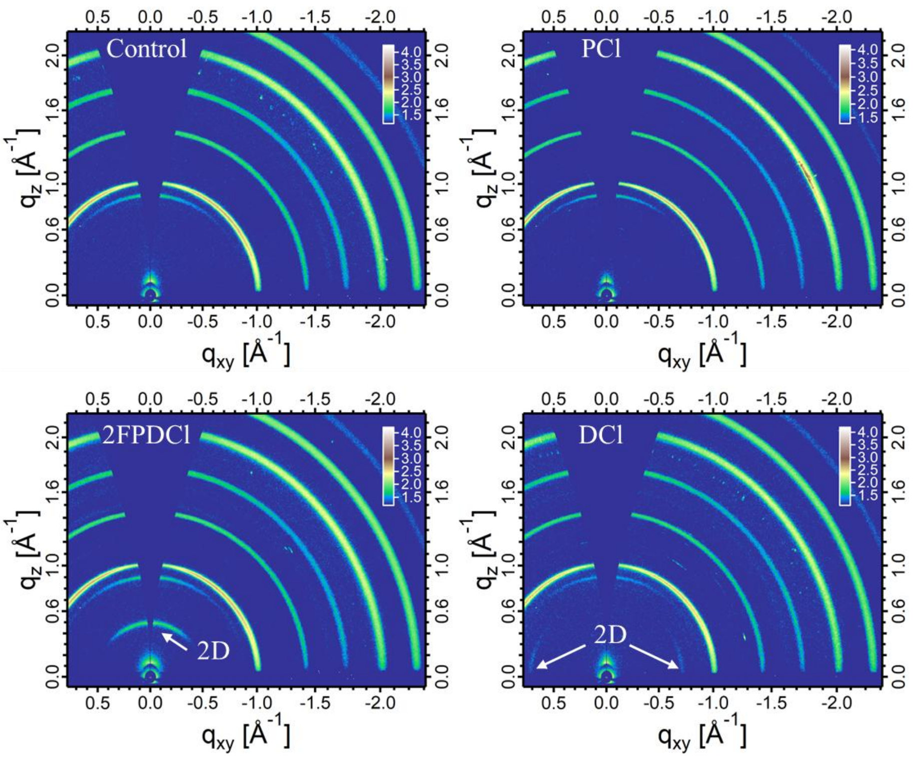

Supplementary Fig. 10 2D GIWAXS patterns of the control, PCl-treated, 2FPDCl-treated and DCl-treated perovskite films with a grazing incidence angle of 0.2°. Careful observation, reveals a new signal at $q_z = 0.49$ Å^{-1} in only the 2FPDCl-treated sample and a new signal at $q_{xy} = 0.72$ Å^{-1} in only the DCl-treated sample. Evidently, the crystallographic orientation of the 2D phase at the DCl-treated perovskite surface is entirely different from that at the 2FPDCl-treated perovskite surface, indicating that the orientation of 2D perovskite is also determined by the spatial dimensionality of the molecules, which has rarely been highlighted before.

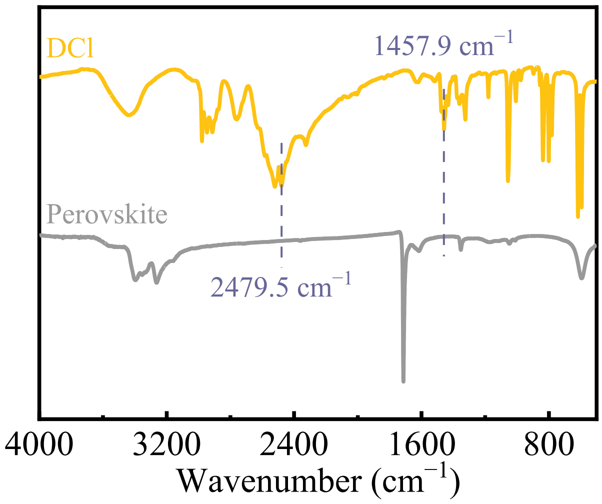

Supplementary Fig. 11 FTIR spectra of the pristine perovskite film and DCl powders. Fourier transform infrared (FTIR) spectroscopy reveals a distinct peak at 1457.9 cm^{-1} for DCl molecules, which serves as a characteristic optical response for detecting their nanoscale distribution in IR mapping profiles.

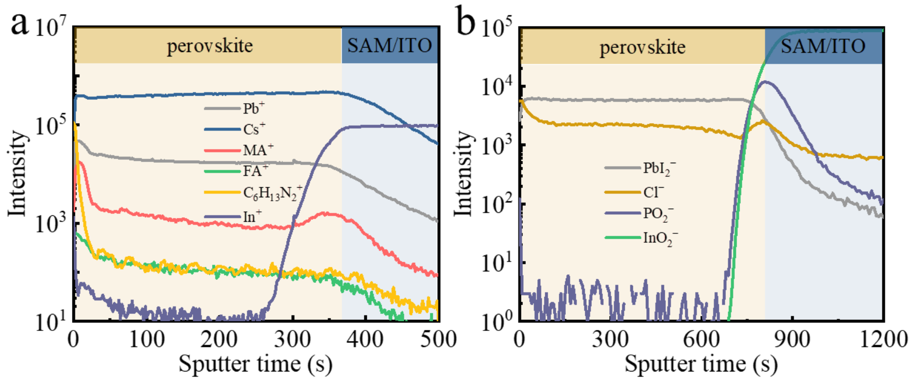

Supplementary Fig. 12 Tof-SIMS 2D depth profiles of DCl-treated perovskite film. (a) Cationic mode. (b) Anionic mode. Pb^+ and PbI_2^- represent the perovskite layer, Cs^+ represents Cesium cations, MA^+ represents Methylammonium cations, FA^+ represents Formamidinium cations, $C_6H_{13}N_2^+$ represents cage-like diammonium cations, Cl^- represents chloride anions, PO_2^- represents the MeO-2PACz self-assembled monolayer, In^+ and InO_2^- represent the indium tin oxide (ITO) substrate.

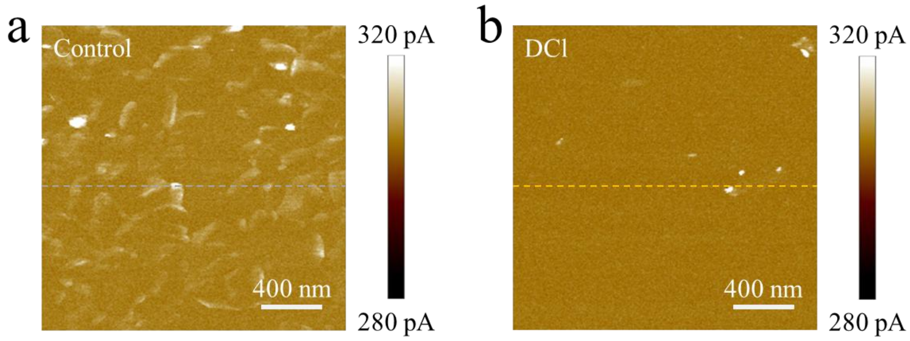

Supplementary Fig. 13 C-AFM images of (a) control and (b) DCl-treated perovskite films.

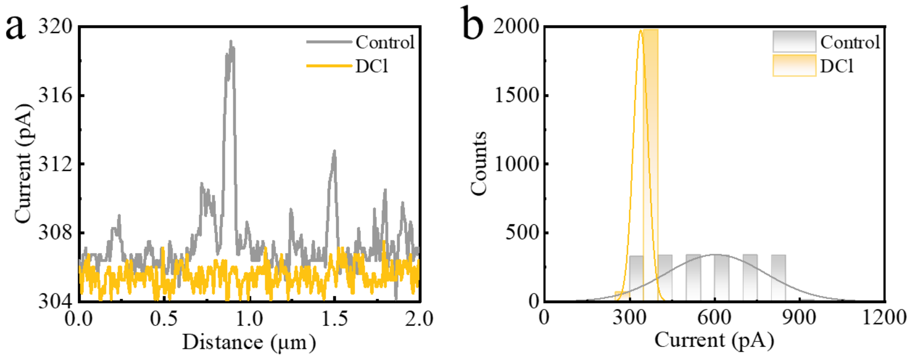

Supplementary Fig. 14 (a) Line profiles of local current flow for the gray and yellow dotted lines in the c-AFM images (Supplementary Fig. 13). (b) Statistical plots of the current extracted from the C-AFM images for the control and DCl-treated perovskite films.

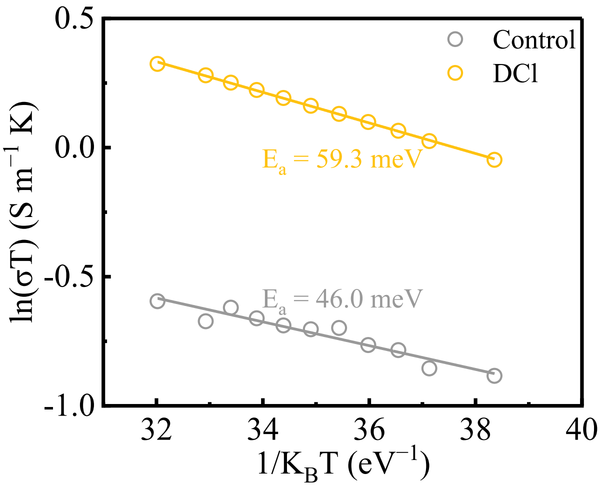

Supplementary Fig. 15 Temperature-dependent conductivity of control and DCl-treated perovskite films. The activation energy value of the control perovskite is determined to be 46 meV, whereas for the DCl-treated film, it increases to 59.3 meV. This result indicates that DCl have a substantially boosted energy barrier for ion migration.

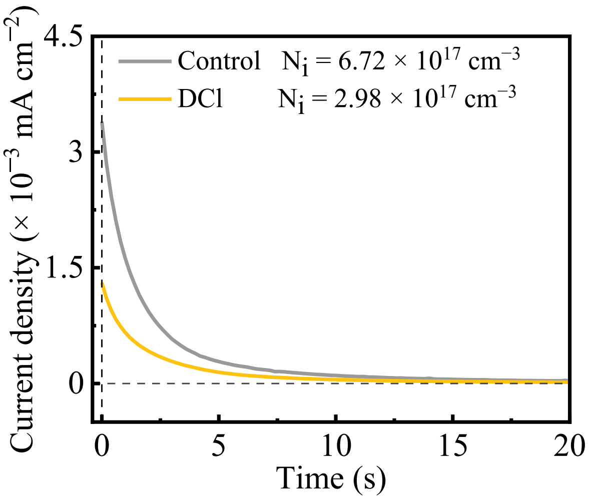

Supplementary Fig. 16 Transient ionic currents (TIC) for extracting mobile ion concentration within the control and DCl-treated perovskite films. We estimated the concentration of mobile ions using a mobile ion charging – discharging method, revealing a decrease from 6.72×10^{17} cm^{-3} in the control device to 2.98×10^{17} cm^{-3} in DCl-treated device. The mobile ion concentration in the DCl-treated device is approximately two times lower than that in the control device as the result of enhanced defect passivation after DCl incorporation. The decreased mobile ion concentration, combined with the increased ion migration activation energy (Supplementary Fig. 15) are conducive to suppressing phase segregation and enhancing device stability.

The concentration of mobile ions in the perovskite film was estimated from the TIC measurement based on the following formula[4]:

$$n=\frac{\int_{t_1}^{t_2} J\,dt}{eL}$$

where t is the time, J is the current density, e is the elementary charge and L is the thickness of the perovskite film. The transient ionic relaxation current was measured in the dark under an external forward bias at V_{OC} for 60 seconds. The observed current primarily originates from the redistribution of mobile ions within the perovskite layer after the removal of the applied voltage, as dictated by the dark test conditions.

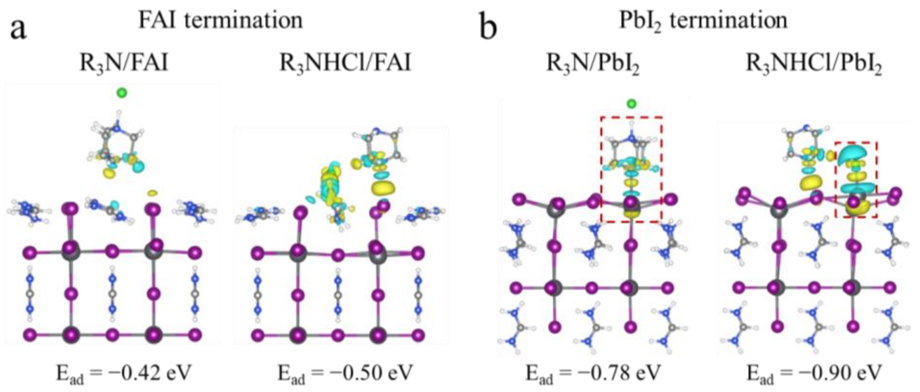

Supplementary Fig. 17 DFT modeling of the interaction of the DCl with (a) FAI-terminated and (b) PbI_2 terminated (100) perovskite surfaces. White, light gray, blue, violet, green and gray balls represent the H, C, N, I, Cl and Pb atoms, respectively. Charge density difference: Yellow iso-surface signifies electron accumulation, while blue represents electron depletion.

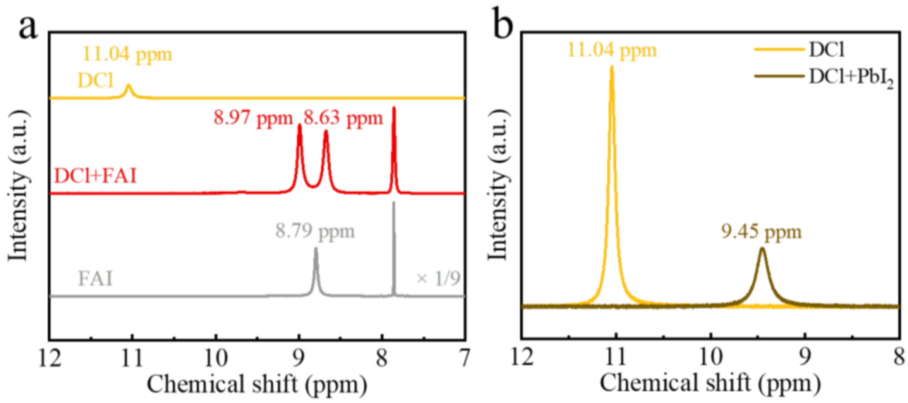

Supplementary Fig. 18 (a) ^1H nuclear magnetic resonance (NMR) spectra of DCl, FAI and DCl+FAI. (b) ^1H nuclear magnetic resonance (NMR) spectra of DCl and DCl+PbI_2. Liquid-state ^1H nuclear magnetic resonance (^1H NMR) was carried out to further explore the interaction mechanism of DCl with perovskite. The chemical shift at 11.04 ppm corresponds to the R_3NH^+ in DCl. Upfield shift trends of ^1H signal for the R_3NH^+ group in DCl was found when mixing DCl with FAI, as depicted in Supplementary Fig. 18a. ^1H signal of the R_3NH^+ group in DCl moves from 11.04 ppm to 9.68 ppm. In addition, the resonance signal (8.79 ppm) of protonated ammonium in FAI splits into two peaks at 8.97 ppm and 8.63 ppm, respectively. These chemical shifts indicate the formation of hydrogen bonding between DCl and FAI[5]. Upon addition of PbI_2 to DCl, ^1H signal of the R_3NH^+ group shows an obviously upfield shift from 11.04 ppm to 9.45 ppm (Supplementary Fig. 18b), which is attributed to the shielding effect induced by anion exchange from Pb-I bond to Pb-Cl bond and the direct ionic binding of Cl^- with the undercoordinated Pb^{2+}[6].

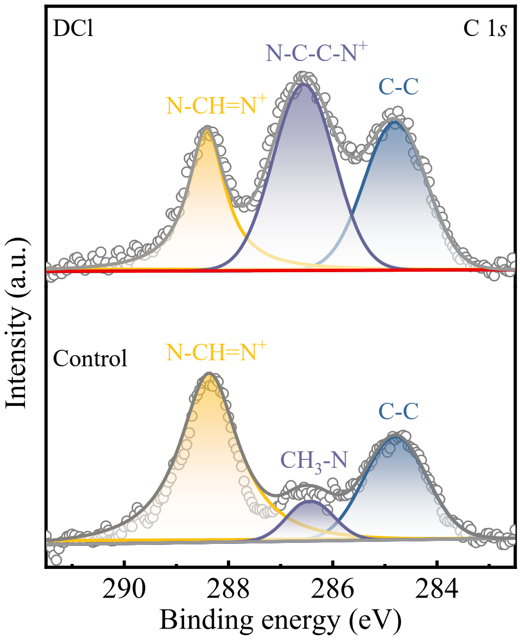

Supplementary Fig. 19 C 1*s* spectra of the control and DCl-treated perovskite films. In the C 1*s* XPS core (Supplementary Fig. 19), the binding energies centered at ~284.8, 286.6, and 288.4 eV are assigned to C-C, N-C-C-N, and N-C=N, respectively.

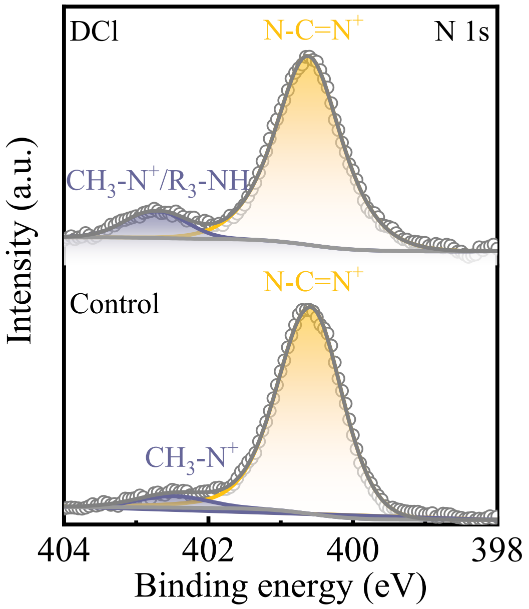

Supplementary Fig. 20 N 1*s* spectra of the control and DCl-treated perovskite films. An obvious increase in the higher binding energy peak at 402.7 eV is detected, likely corresponding to R_3-N bond. The obvious N-C-C-N (Supplementary Fig. 19) and R_3-N (Supplementary Fig. 20) characteristic peaks for DCl-treated film collectively indicates the existence of cage-like diammonium cation on the perovskite surface and its dominant interaction with the perovskite.

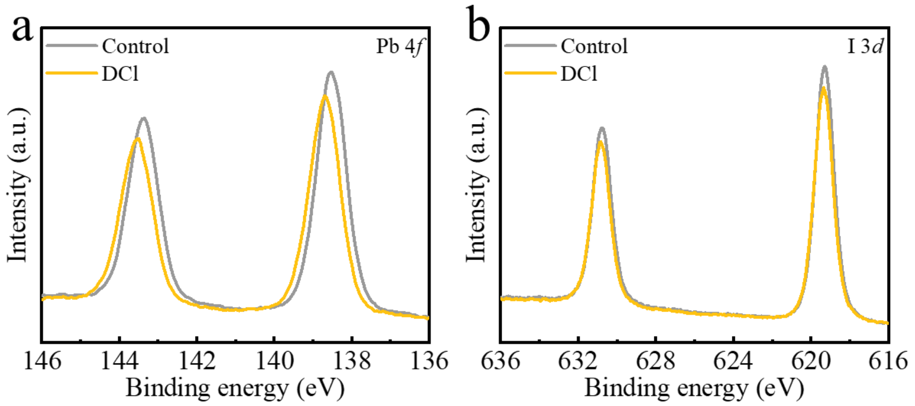

Supplementary Fig. 21 (a) Pb 4*f* XPS spectra of the control and DCl-treated perovskite films. (b) I 3*d* XPS spectra of the control and DCl-treated perovskite films. We notice that with the incorporation of DCl, the characteristic peaks in the Pb 4*f* XPS spectra shift toward the higher binding energy (from 143.37 and 138.51 eV for $4f_{5/2}$ and $4f_{7/2}$ to 143.55 and 138.66 eV, respectively), which may originate from the bond formation between the uncoordinated Pb^{2+} in perovskite or PbI_2 and DCl (Supplementary Fig. 21 and Supplementary Table. 1). Similar upward shift of the corresponding characteristic peaks in the I 4*d* XPS spectra is also observed (Supplementary Fig. 21b and Supplementary Table. 2), in which I $3d_{3/2}$ (630.71 eV) and I $3d_{5/2}$ (619.26 eV) for the control film slightly shift toward higher binding energy position of 630.77 and 619.31 eV, respectively, after DCl post-treatment, which could be ascribed to the incorporation of Cl[7].

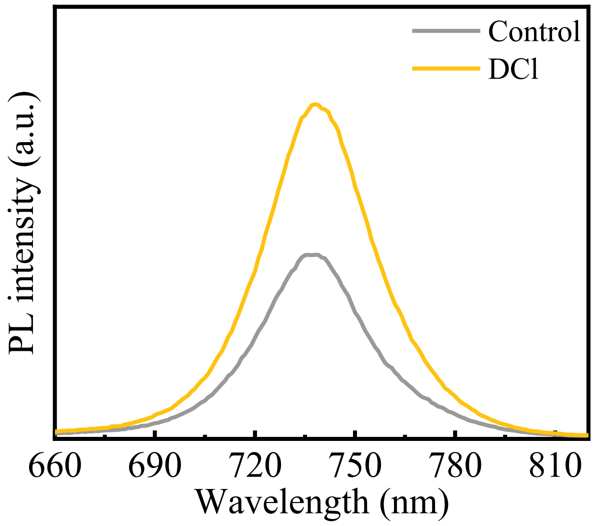

Supplementary Fig. 22 Steady-state PL spectra of the control and DCl-treated perovskite films.

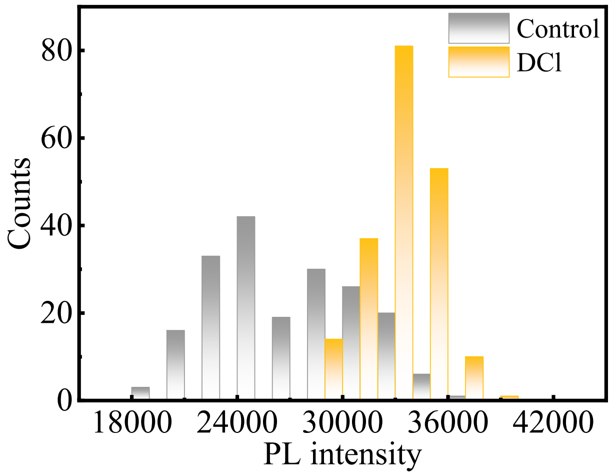

Supplementary Fig. 23 Histogram of PL intensity of the control and DCl-treated perovskite films.

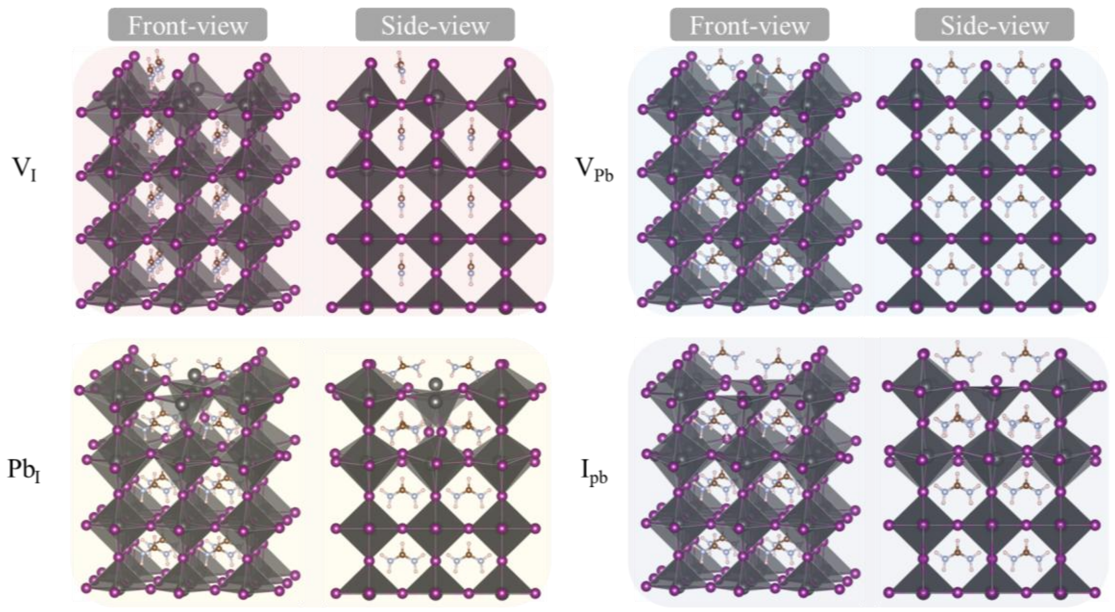

Supplementary Fig. 24 Front-view and side-view of four prevalent surface defects: V_I, Pb_I, V_{Pb} and I_{Pb}. Pink, light brown, light gray, violet and gray balls represent the H, C, N, I and Pb atoms, respectively.

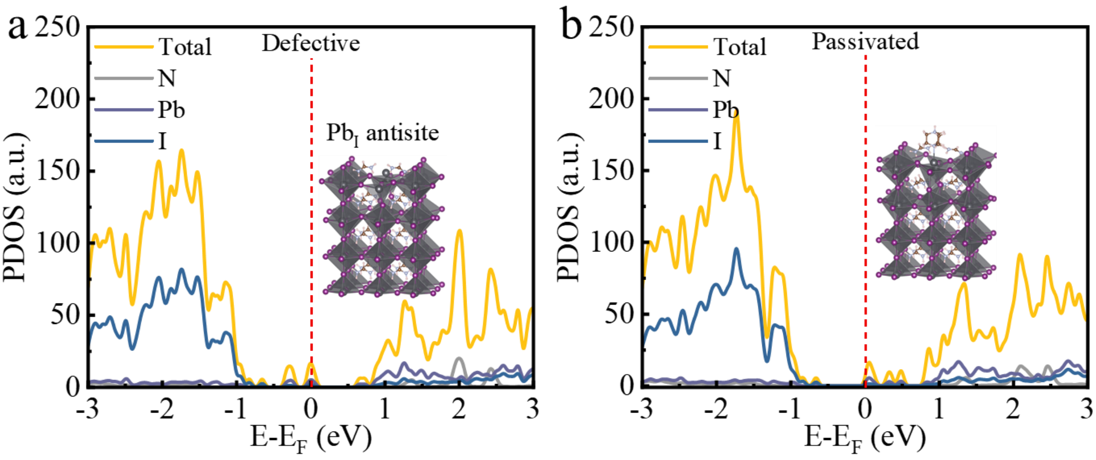

Supplementary Fig. 25 The partial density of states (PDOSs) of (a) defective perovskite (Pb_I antisite) and (b) passivated perovskite by DCl.

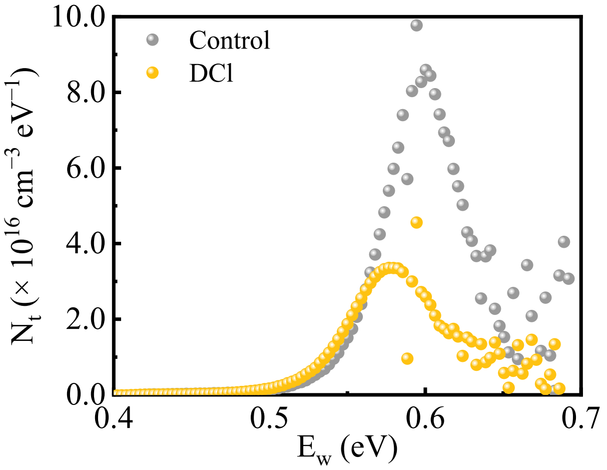

Supplementary Fig. 26 Trap density of states (tDOS) spectra of the control and DCl-treated devices.

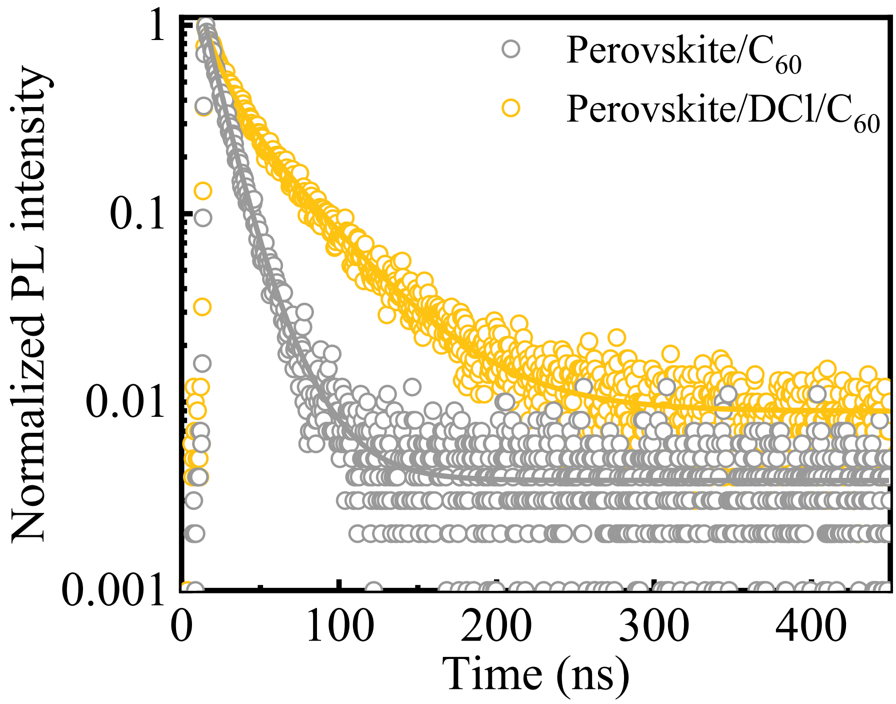

Supplementary Fig. 27 TRPL decay curves of the control and DCl-treated perovskite films capped with a C_{60} layer.

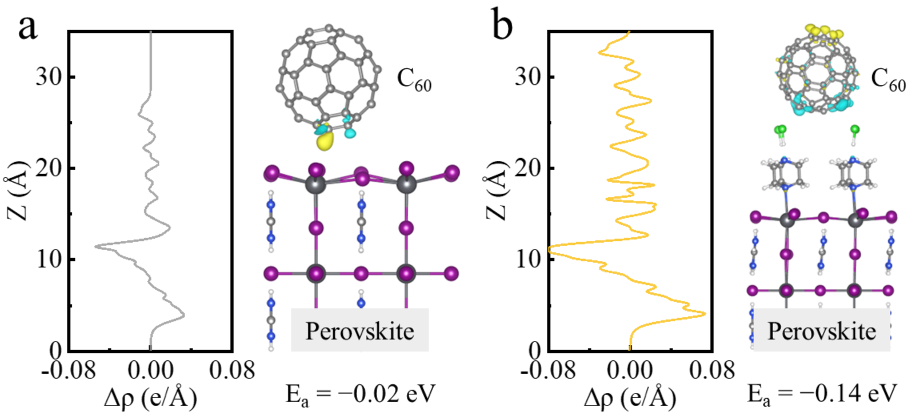

Supplementary Fig. 28 Charge density difference (yellow, accumulation; blue, depletion) along with the corresponding plane-averaged profiles for C_{60} ETL anchoring onto both the (a) control and (b) DCl passivated (100) perovskite surfaces. The calculated adsorption energy (E_a) is also shown. White, light gray, blue, violet, green and gray balls represent the H, C, N, I, Cl and Pb atoms, respectively.

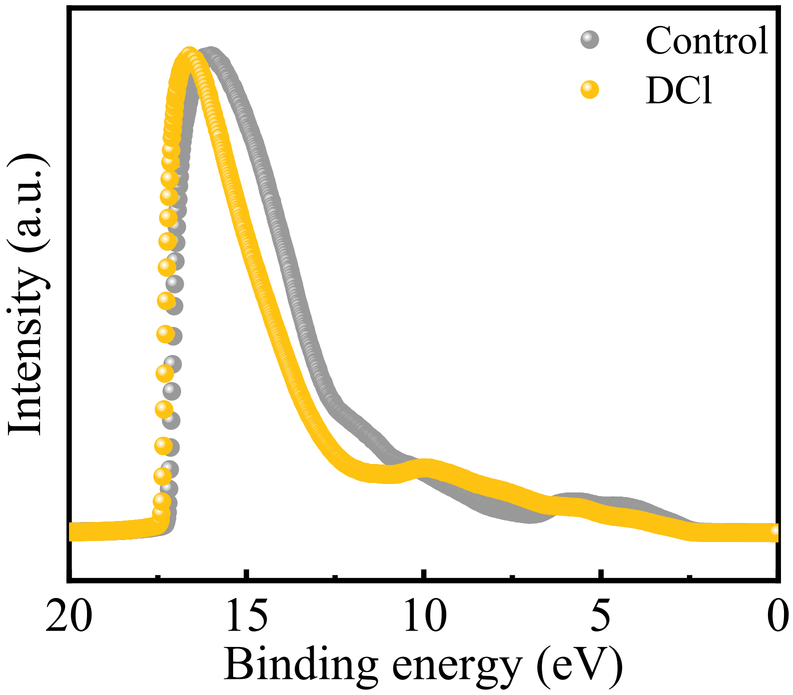

Supplementary Fig. 29 UPS spectra of the control and DCl-treated perovskite films.

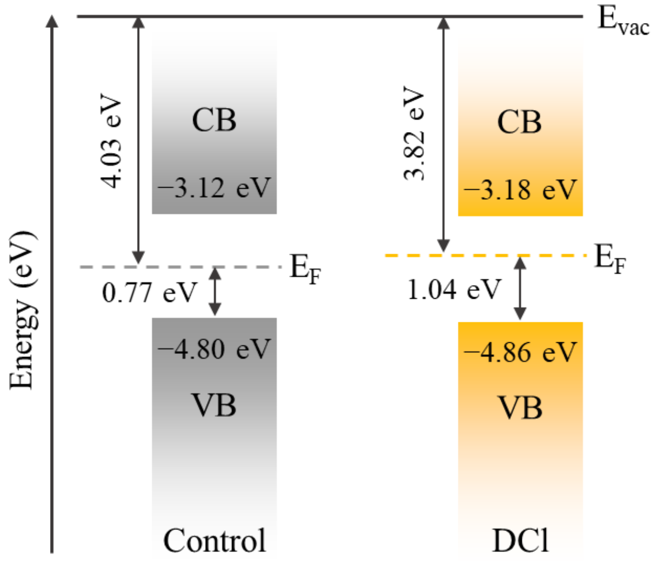

Supplementary Fig. 30 Energy-level scheme based on the UPS spectra.

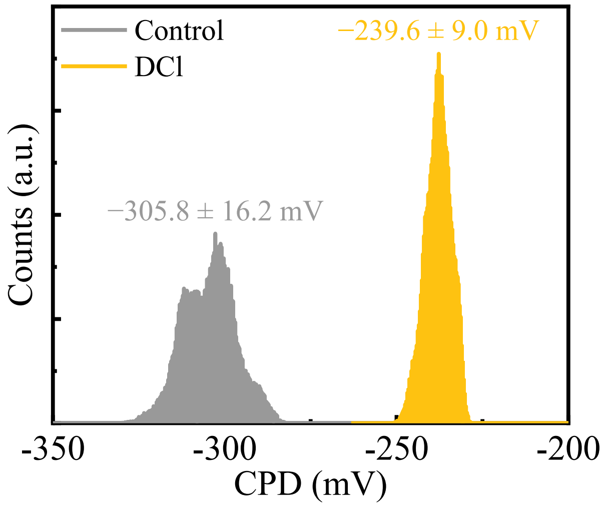

Supplementary Fig. 31 CPD statistical distributions derived from the KPFM images of the control and DCl-treated perovskite films.

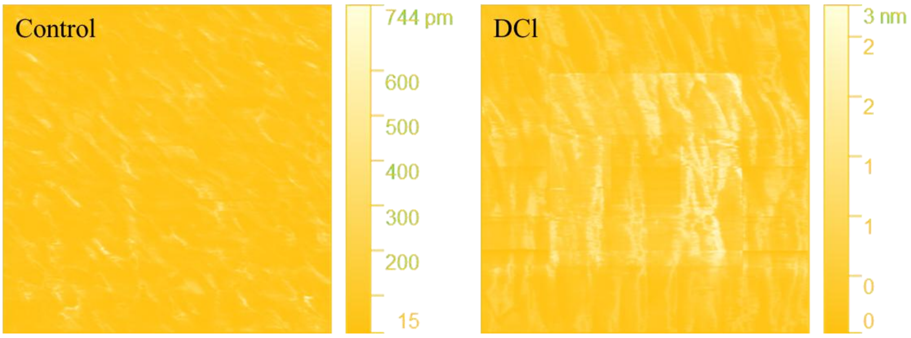

Supplementary Fig. 32 PFM amplitude images of the control and DCL-treated perovskite films.

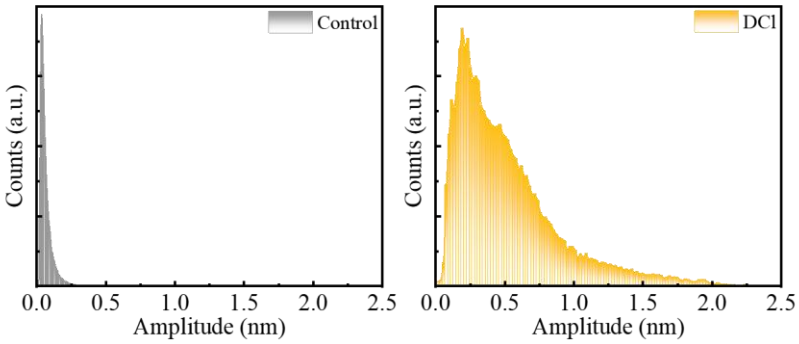

Supplementary Fig. 33 The corresponding amplitude distribution histogram of the control and DCl-treated perovskite films.

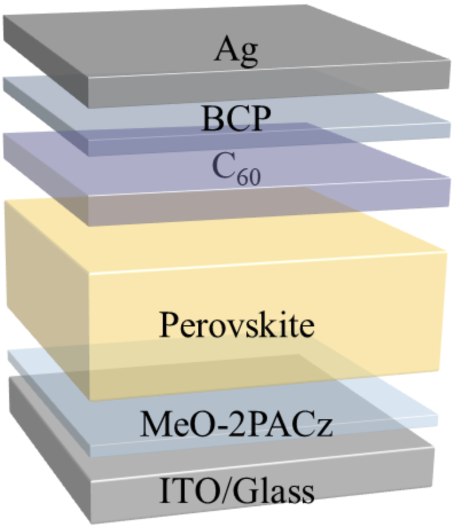

Supplementary Fig. 34 Device architecture of the single-junction perovskte solar cells.

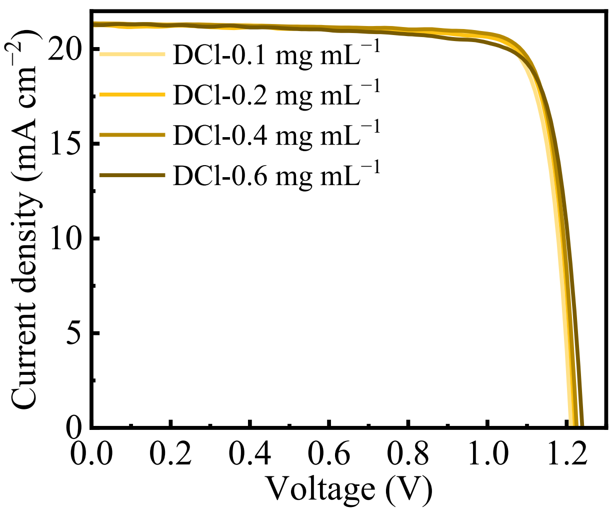

Supplementary Fig. 35 *J*–*V* curves of champion devices treated with 0.1, 0.2, 0.4 and 0.6 mg mL^{-1} of DCl.

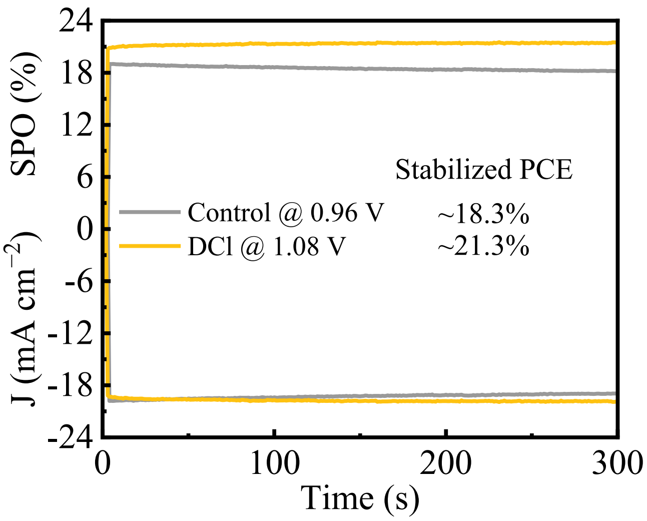

Supplementary Fig. 36 Steady-state output of PCEs for the champion control and DCl-treated perovskite solar cells.

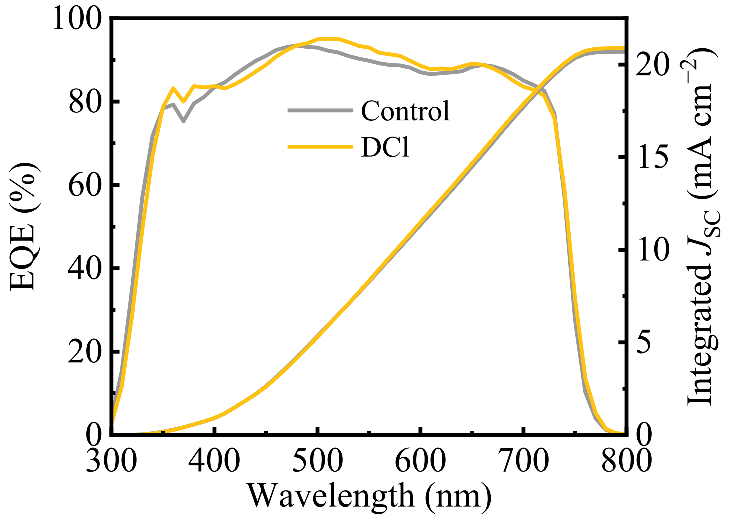

Supplementary Fig. 37 EQE spectra of the control and DCl-treated perovskite solar cells.

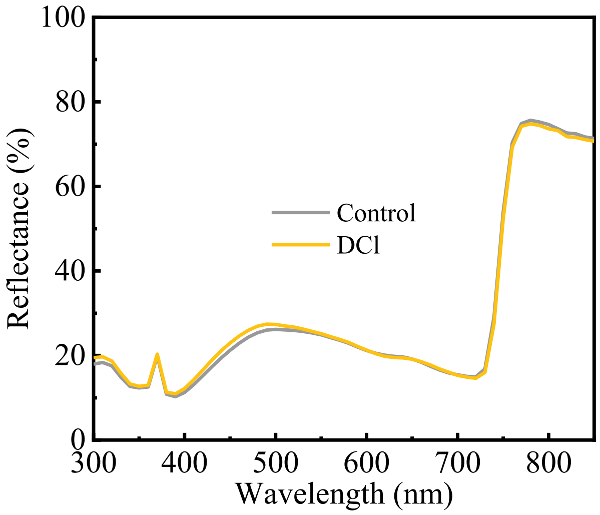

Supplementary Fig. 38 Reflectance spectra of the control and DCl-treated devices.

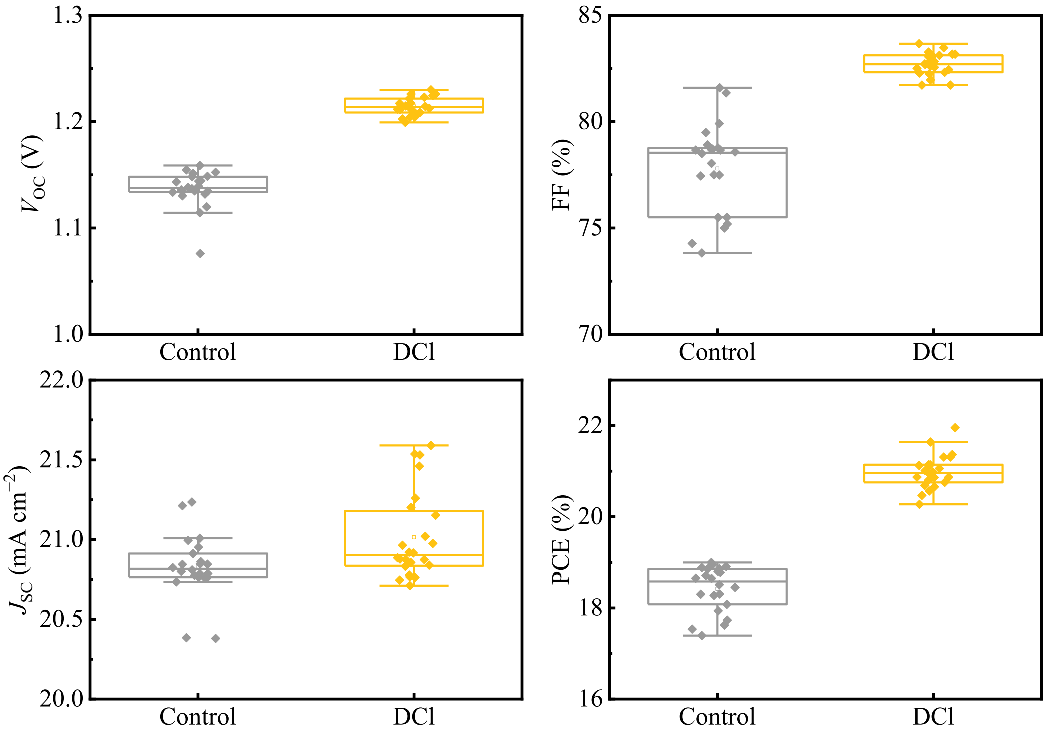

Supplementary Fig. 39 Statistical distributions of PV parameters for control and DCl-treated perovskite solar cells.

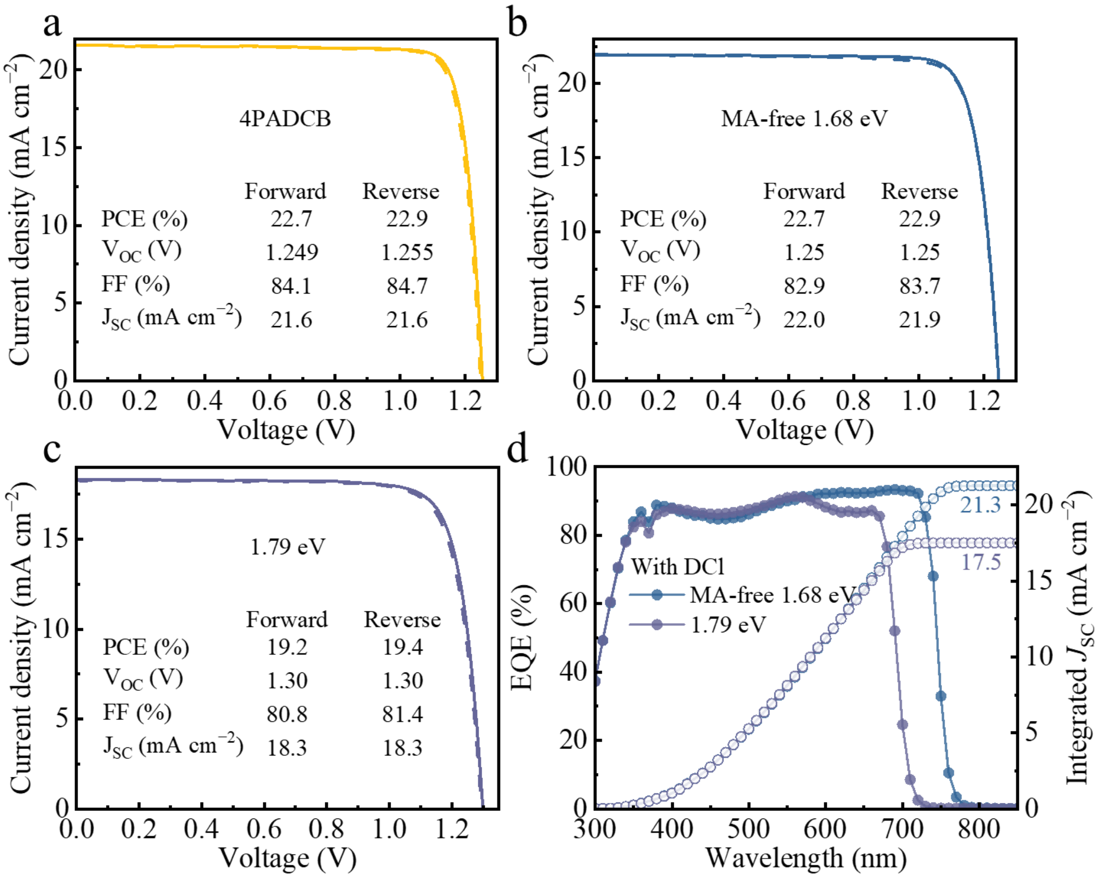

Supplementary Fig. 40 (a) *J–V* curves of the DCl-treated perovskite solar cells based on 4PADCB hole transport layer. (b, c) *J–V* curves and (d) EQE spectra and the corresponding integrated J_{SC}s for 1.68 eV MA-free and 1.79 eV wide-bandgap perovskite solar cells.

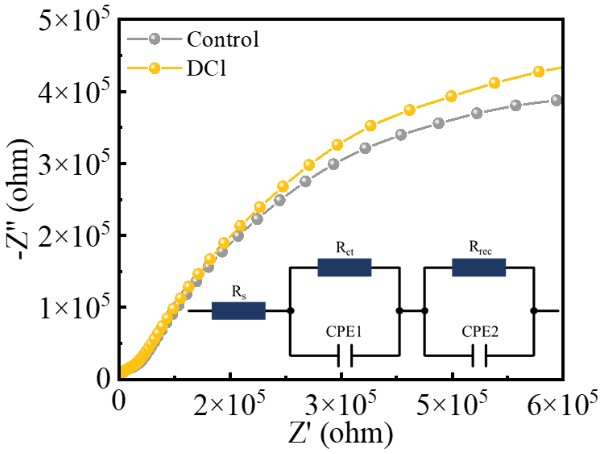

Supplementary Fig. 41 EIS spectra of the control and DCl-treated devices (inset: equivalent circuit).

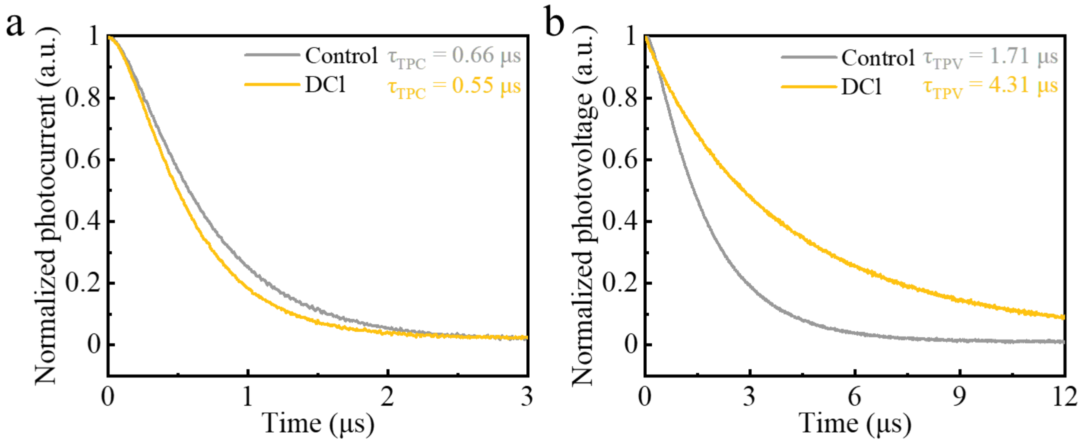

Supplementary Fig. 42 (a) TPC and (b) TPV decay curves of the control and DCl-treated devices.

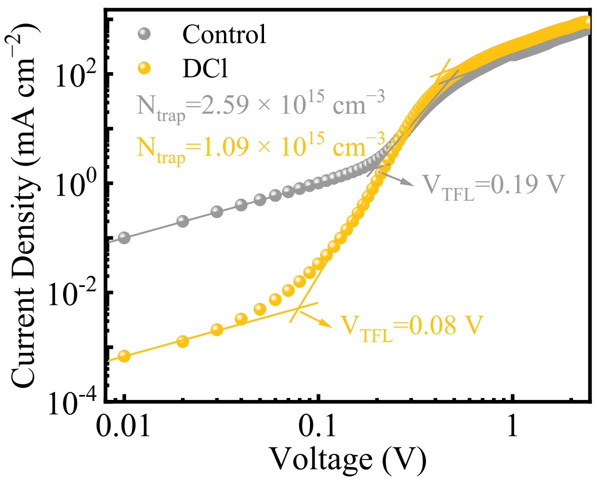

Supplementary Fig. 43 Dark J–V curves of electron-only devices (Glass/ITO/ALD-SnO_X/perovskite/C_{60}/BCP/Ag) with and without DCl treatment. The defect state density (Ntrap) can be estimated using the following equation:

$$N_{trap}=\frac{2\varepsilon_r\varepsilon_0 V_{TFL}}{2qL^2}$$

Where q is the elementary charge, V_{TFL} is the trap filling limit voltage, ε_r signifies the relative dielectric constant of the perovskite, and ε_0 is the vacuum dielectric constant. L represents the perovskite film thickness.

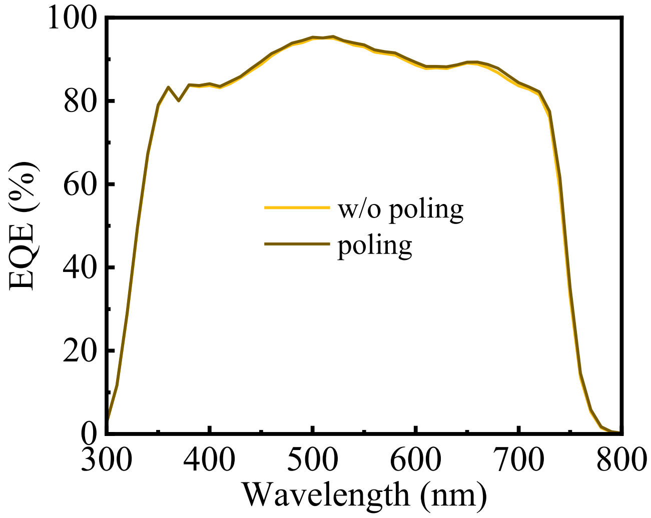

Supplementary Fig. 44 EQE spectra of the DCl-treated perovskite solar cells with and without poling (20.9 mA cm^{-2} without poling and 21.1 mA cm^{-2} with poling).

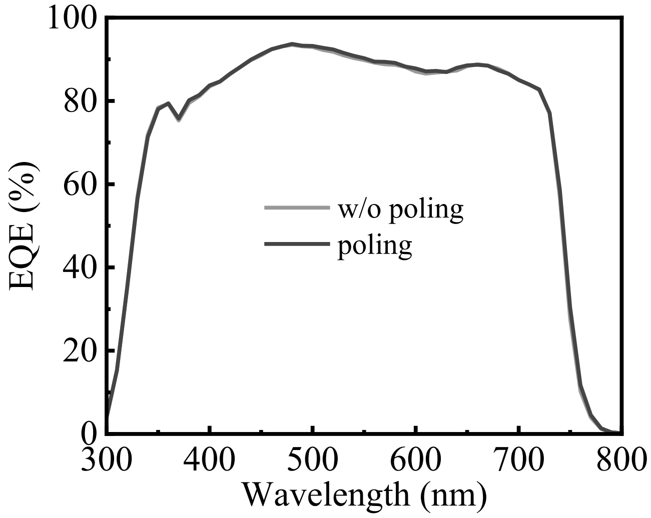

Supplementary Fig. 45 EQE spectra of the control perovskite solar cells with and without poling (20.7 mA cm^{-2} without poling and 20.8 mA cm^{-2} with poling).

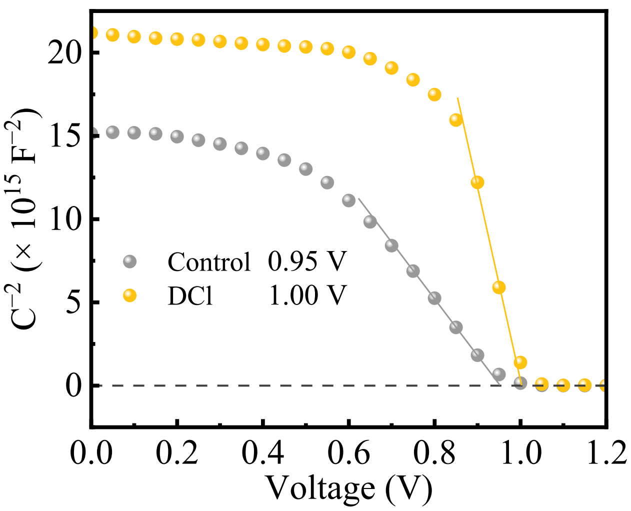

Supplementary Fig. 46 Mott–Schottky plots of the control and DCl-treated perovskite solar cells. The curves on top of the data were obtained by linear fitting the drop region of Mott–Schottky plots, and the built-in electric field was extracted via the intercept of the straight line with the x axis.

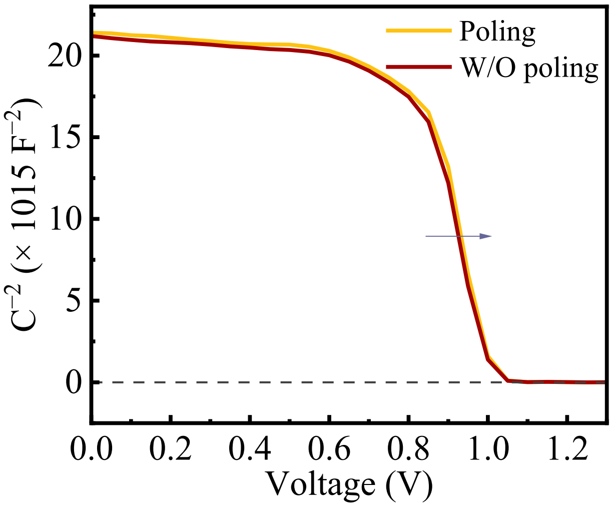

Supplementary Fig. 47 Mott–Schottky plots of the DCl-treated perovskite solar cell before and after poling.

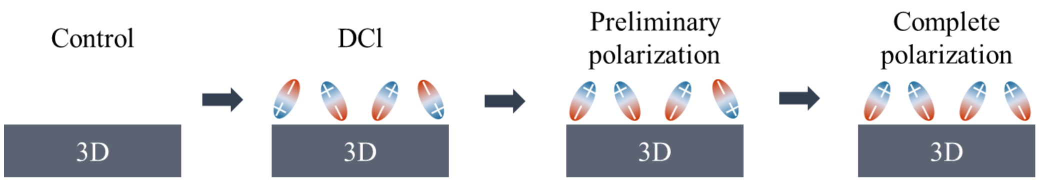

Supplementary Fig. 48 Schematic illustration of electric dipole switching under external polarization in the DCl-treated perovskite surface.

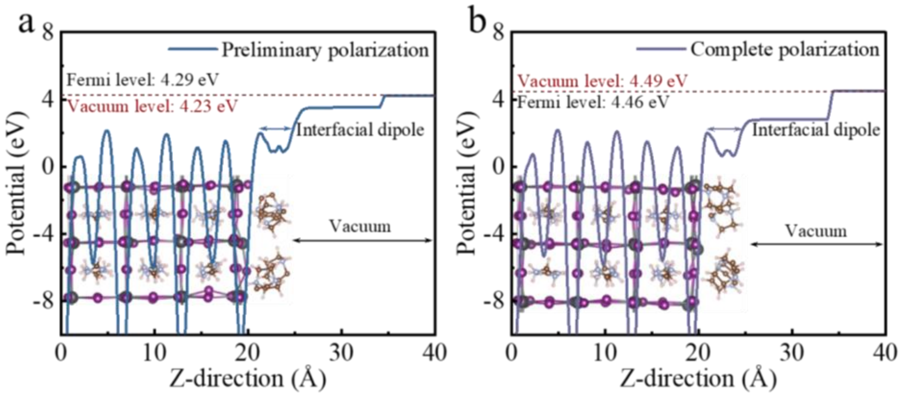

Supplementary Fig. 49 DFT-calculated electrostatic potential and surface work functions of perovskites the adsorption of cage-like cations. (a) Preliminary polarization, (b) Complete polarization.

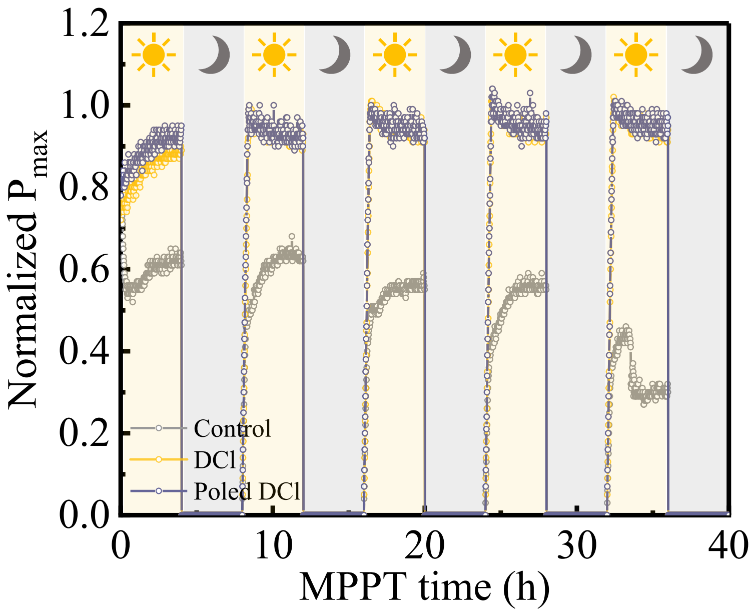

Supplementary Fig. 50 Operational stability of control, DCl-treated and poled DCl-treated devices by MPP tracking with an artificially induced day-night cycle. As presented in Supplementary Fig. 50, the control device shows a continuous drop in maximum power point (P_{max}) without any recovery after storage in the dark, indicating poor stability. In addition, the control device consistently exhibits a gradual increase in P_{max} during each light cycle, which is detrimental to achieving optimal energy output under realistic light-dark cycling conditions. In contrast, the DCl-treated and poled DCl-treated devices maintain stable performance without noticeable degradation during 40 hours of day-night cycle testing. In case of DCl-treated and poled DCl-treated devices, although the P_{max} drops to 90-95% of its initial value for each light cycle of 4 hours, it fully recovers to its original level after a subsequent 4-hour dark period. This reversible behavior demonstrates a self-healing effect, which could be practically beneficial for commercial perovskite solar cell operation, as it enables nighttime self-recovery from the ~5-10% photodegradation accumulated during daytime use[8]. Moreover, in each light cycle, the P_{max} values of the DCl-treated and poled DCl-treated devices immediately reach their optimal levels at the onset of illumination, which is favorable for maximizing energy output under realistic light-dark cycling conditions. Notably, the poled DCl-treated device delivers a higher output power in the initial light cycle but converges to the same performance level as the DCl-treated device in the following cycles, indicating that the ferroelectric effect contributes to a slight, yet short-lived, enhancement in energy yield. This behavior may be ascribed to the transient ferroelectric characteristic of the DCl-mediated quasi-2D perovskite. In fact, a permanent ferroelectric state would be energetically unfavorable for 2D perovskites, as it would inevitably compromise the structural stability of the 2D perovskite. Before poling, the ferroelectric organic cations predominantly occupy the A-sites to maintain the structural integrity of the quasi-2D lattice. Upon applying an external electric field, these cations undergo ferroelectric switching and deviate from their equilibrium positions, generating an internal depolarization field within the ferroelectric spacer layers that facilitates charge transport. However, during the dark period, the organic

cations gradually return to their original A-site locations to restore the structural stability of the 2D perovskite. This dynamic process may explain why the poled DCl-treated device delivers enhanced output power during the initial light cycle but converges to the same performance level as the unpoled DCl-treated device in subsequent cycles.

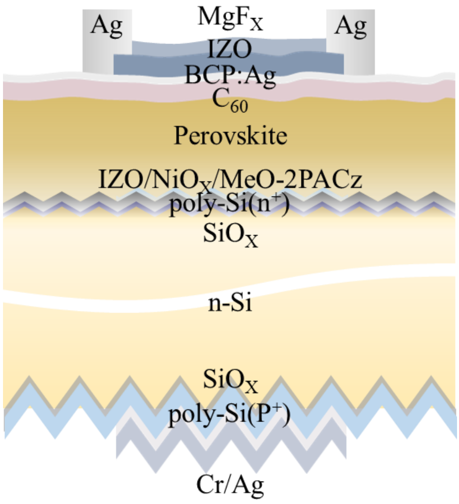

Supplementary Fig. 51 Schematic illustration of the perovskite/silicon tandem solar cell architecture.

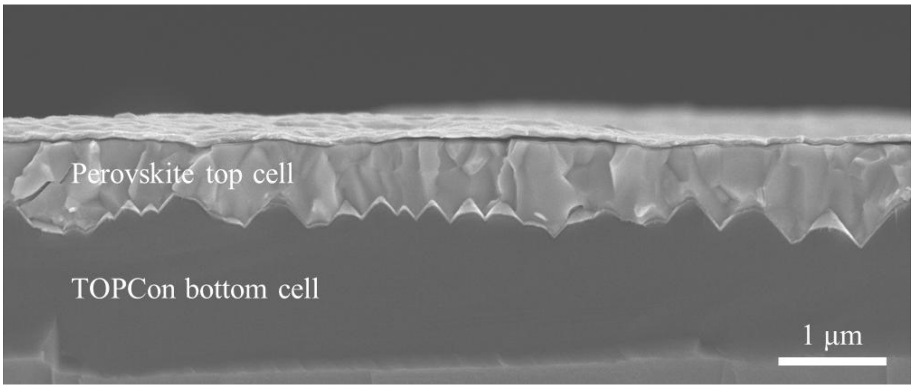

Supplementary Fig. 52 Cross-sectional SEM image of the DCl-treated perovskite/silicon tandem solar cell.

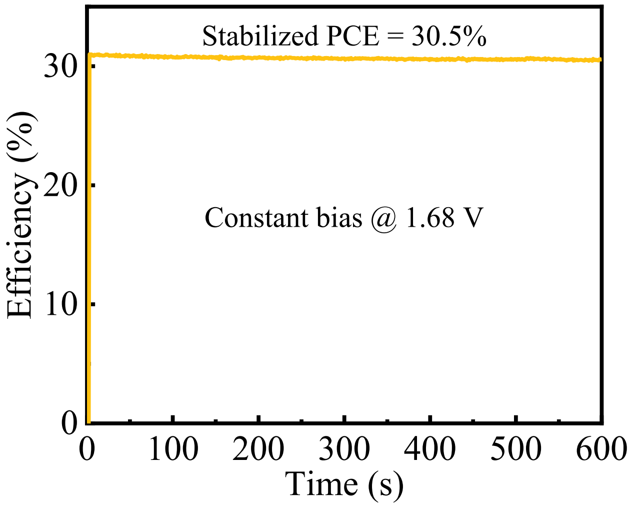

Supplementary Fig. 53 Steady state PCE of the champion DCl-treated perovskite/silicon TSC under 100 mW cm^{-2} AM1.5G illumination.

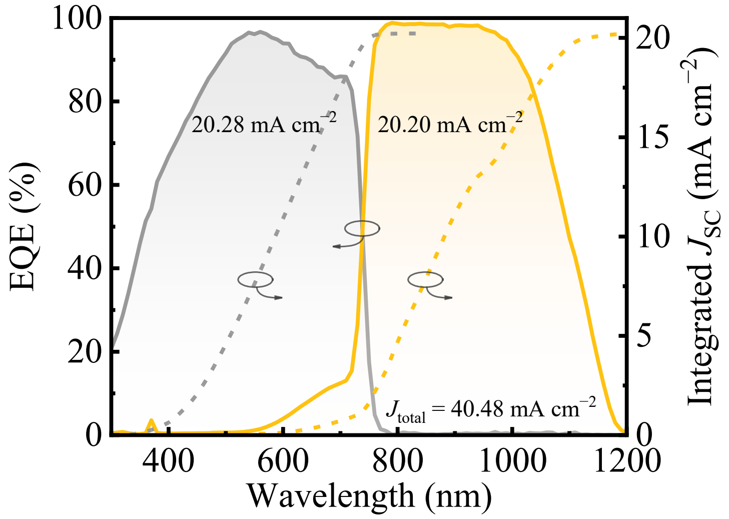

Supplementary Fig. 54 EQE spectrum of the DCl-treated perovskite/silicon tandem solar cell.

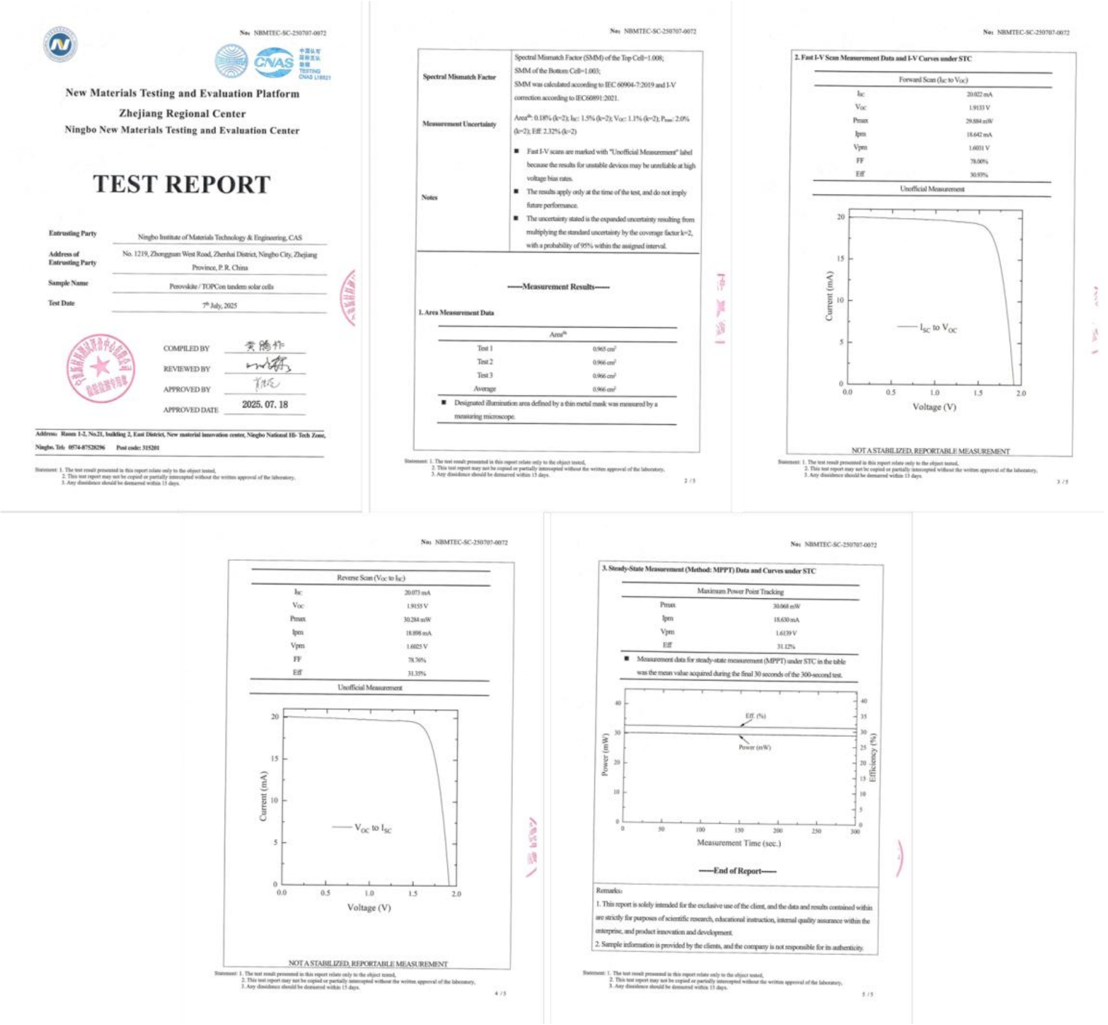

No: NBMTEC-SC-250707-0072

New Materials Testing and Evaluation Platform

Zhejiang Regional Center

Ningbo New Materials Testing and Evaluation Center

TEST REPORT

**Entrusting Party**	Ningbo Institute of Materials Technology & Engineering, CAS
**Address of Entrusting Party**	No. 1219, Zhongguan West Road, Zhenhai District, Ningbo City, Zhejiang Province, P. R. China
**Sample Name**	Perovskite / TOPCon tandem solar cells
**Test Date**	7th July, 2025

COMPILED BY

REVIEWED BY

APPROVED BY

APPROVED DATE 2025. 07. 18

Address: Room 1-2, No.21, building 2, East District, New material innovation center, Ningbo National Hi- Tech Zone, Ningbo. Tel: 0574-87528296 Post code: 315201

Statement: 1. The test result presented in this report relate only to the object tested.
2. This test report may not be copied or partially intercepted without the written approval of the laboratory.
3. Any dissidence should be demurred within 15 days.

No: NBMTEC-SC-250707-0072

**Spectral Mismatch Factor**	Spectral Mismatch Factor (SMM) of the Top Cell=1.008; SMM of the Bottom Cell=1.003; SMM was calculated according to IEC 60904-7:2019 and I-V correction according to IEC60891:2021.
**Measurement Uncertainty**	Area[th]: 0.18% (k=2); I_{sc}: 1.5% (k=2); V_{OC}: 1.1% (k=2); P_{max}: 2.0% (k=2); Eff: 2.32% (k=2)
**Notes**	■ Fast I-V scans are marked with "Unofficial Measurement" label because the results for unstable devices may be unreliable at high voltage bias rates. ■ The results apply only at the time of the test, and do not imply future performance. ■ The uncertainty stated is the expanded uncertainty resulting from multiplying the standard uncertainty by the coverage factor k=2, with a probability of 95% within the assigned interval.

-----Measurement Results-----

1. Area Measurement Data

	Area[th]
Test 1	0.965 cm^2
Test 2	0.966 cm^2
Test 3	0.966 cm^2
Average	0.966 cm^2

■ Designated illumination area defined by a thin metal mask was measured by a measuring microscope.

Statement: 1. The test result presented in this report relate only to the object tested.
2. This test report may not be copied or partially intercepted without the written approval of the laboratory.
3. Any dissidence should be demurred within 15 days.

2 / 5

No: NBMTEC-SC-250707-0072

2. Fast I-V Scan Measurement Data and I-V Curves under STC

	Forward Scan (I_{sc} to V_{oc})
Isc	20.022 mA
Voc	1.9133 V
Pmax	29.884 mW
Ipm	18.642 mA
Vpm	1.6031 V
FF	78.00%
Eff	30.93%

Unofficial Measurement

NOT A STABILIZED, REPORTABLE MEASUREMENT

Statement: 1. The test result presented in this report relate only to the object tested.
2. This test report may not be copied or partially intercepted without the written approval of the laboratory.
3. Any dissidence should be demurred within 15 days.

3 / 5

No: NBMTEC-SC-250707-0072

	Reverse Scan (V_{oc} to I_{sc})
Isc	20.073 mA
Voc	1.9155 V
Pmax	30.284 mW
Ipm	18.898 mA
Vpm	1.6025 V
FF	78.76%
Eff	31.35%

Unofficial Measurement

NOT A STABILIZED, REPORTABLE MEASUREMENT

Statement: 1. The test result presented in this report relate only to the object tested.
2. This test report may not be copied or partially intercepted without the written approval of the laboratory.
3. Any dissidence should be demurred within 15 days.

4 / 5

No: NBMTEC-SC-250707-0072

3. Steady-State Measurement (Method: MPPT) Data and Curves under STC

	Maximum Power Point Tracking
Pmax	30.068 mW
Ipm	18.630 mA
Vpm	1.6139 V
Eff	31.12%

■ Measurement data for steady-state measurement (MPPT) under STC in the table was the mean value acquired during the final 30 seconds of the 300-second test.

-----End of Report-----

Remarks:

1. This report is solely intended for the exclusive use of the client, and the data and results contained within are strictly for purposes of scientific research, educational instruction, internal quality assurance within the enterprise, and product innovation and development.
2. Sample information is provided by the clients, and the company is not responsible for its authenticity.

Statement: 1. The test result presented in this report relate only to the object tested.
2. This test report may not be copied or partially intercepted without the written approval of the laboratory.
3. Any dissidence should be demurred within 15 days.

5 / 5

Supplementary Fig. 55 Certificated results of DCl-treated perovskite/silicon tandem from an accredited photovoltaic certification laboratory.

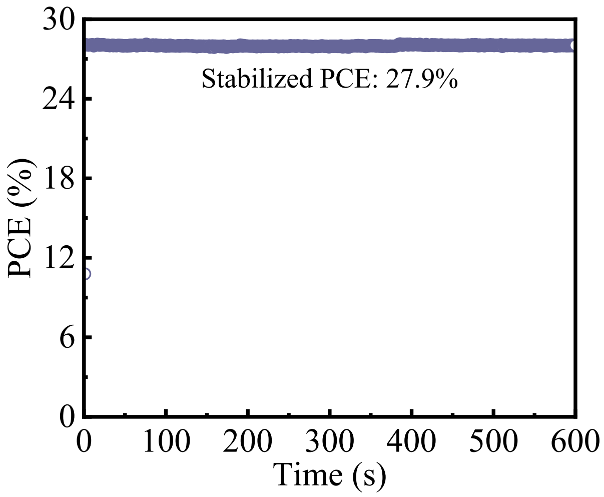

Supplementary Fig. 56 Steady-state PCE with a constant bias of 1.58 V for the large-area monolithic perovskite/silicon TSCs with an active area of 4 cm^2.

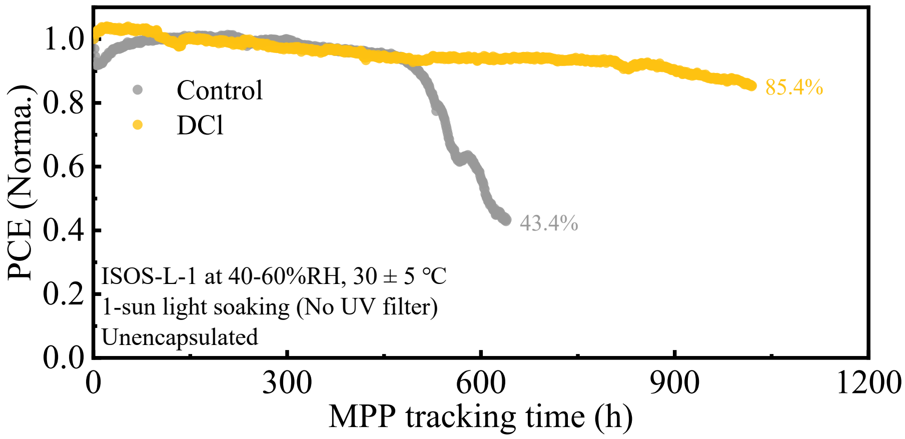

Supplementary Fig. 57 Continuous MPP tracking of the unencapsulated control and DCl-treated perovskite/silicon TSCs under 1-sun illumination (100 mW cm^{-2}) with a xenon lamp without UV filter in ambient air (30 ± 5 °C, 40–60% RH).

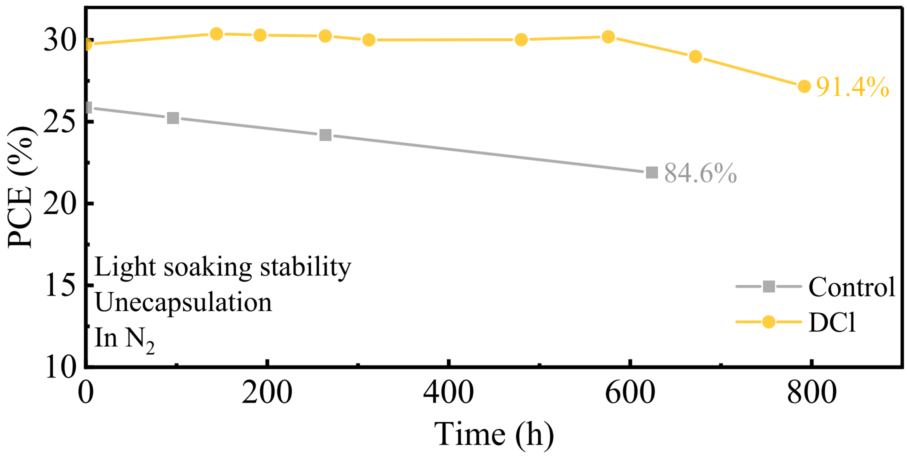

Supplementary Fig. 58 Light soaking stability of unencapsulated control and DCl-treated perovskite/silicon TSCs under 1-sun illumination (LED, 100 mW cm^{-2}) in N_2 atmosphere at open-circuit conditions.

Supplementary Table. 1 Binding energy of Pb 4f XPS spectra for the control and DCl-treated perovskite film.

Pb 4f	Control	DCl
4f 5/2 (eV)	143.37	143.55
4f 7/2 (eV)	138.51	138.66

Supplementary Table. 2 Binding energy of I 3d XPS spectra of the control and DCl-treated perovskite film.

I 3d	Control	DCl
3d 3/2 (eV)	630.71	630.77
3d 5/2 (eV)	619.26	619.31

Supplementary Table. 3 TRPL parameters of the control and DCl-treated perovskite films with a C_{60} layer by the bi-exponential fitting.

Devices	A_1	τ_1 [ns]	A_2	τ_2 [ns]	τ_{avg} [ns]
Perovskite/C_{60}	1.02	10.77	0.17	22.91	12.50
Perovskite/DCl/C_{60}	0.49	10.48	0.52	43.01	27.23

Supplementary Table. 4 Summary of reported perovskite solar cells with ferroelectric materials.

Ferroelectric materials	Structure	Strategy	Ref.
P(VDF-TrFE)	Chain-like	Additive	9
P(VDF-TrFE)	Chain-like	Additive	10
P(VDF-TrFE):DH	Chain-like	Additive	11
R-1-(4-chlorophenyl)ethylamine	Plane-type	Additive	12
Trimethyliodomethylammonium tris(μ2-iodo)-lead	1D	Additive	13
R-1-(4-chlorophenyl)ethylamine	Plane-type	Post-treatment	14
3-pyridinylmethylammonium	Ring-like	Additive	15
4,4-difluoropiperidine hydrochloride	Ring-like	Post-treatment	16
4,4-difluoropiperidine hydrochloride	Ring-like	Post-treatment	17
1,4-diazabicyclo[2.2.2]octane chloride	Cage-like	Post-treatment	This work

Supplementary Table. 5 *J–V* parameters of perovskite single-junction solar cells treated with 0 (control), 0.1, 0.2, 0.4 and 0.6 mg mL^{-1} of DCl.

Concentration [mg mL^{-1}]	V_{OC} [V]	FF [%]	J_{SC} [mA cm^{-2}]	PCE [%]
Control	1.137	78.7	21.2	19.0
0.1	1.211	82.3	21.4	21.3
0.2	1.221	82.7	21.3	21.5
0.4	1.226	83.2	21.5	21.9
0.6	1.240	80.5	21.3	21.3

Supplementary Table. 6 *J–V* parameters of the best control and DCl-treated perovskite solar cells before poling.

W/O poling	V_{OC} [V]	FF [%]	J_{SC} [mA cm^{-2}]	PCE [%]
Control	1.134	78.5	21.2	18.8
	1.137	78.7	21.2	19.0
DCl	1.225	82.9	21.5	21.8
	1.226	83.2	21.5	21.9

Supplementary Table. 7 The average and standard deviation of device parameters for single-junction perovskite solar cells without poling.

Sample	V_{OC} [V]	J_{SC} [mA cm^{-2}]	FF [%]	PCE [%]
Control	1.137±0.017	20.83±0.20	77.80±2.12	18.42±0.50
DCl	1.214±0.004	21.01±0.27	82.70±0.53	20.99±0.38

Supplementary Table. 8 EIS fitting parameters of the control and DCl-treated devices.

Sample	R_s (Ω)	R_{ct} (×10^4 Ω)	R_{rec} (×10^4 Ω)
Control	8.2	97	3.4
DCl	8.1	3.5	86

Supplementary Table. 9 The average and standard deviation of device parameters for single-junction perovskite solar cells after poling.

Sample	V_{OC} [V]	J_{SC} [mA cm^{-2}]	FF [%]	PCE [%]
Control	1.141±0.003	21.04±0.37	78.26±0.57	18.76±0.35
DCl	1.224±0.009	21.53±0.35	83.32±0.63	21.94±0.44

Supplementary Table. 10 *J–V* parameters of the best control and DCl-treated perovskite solar cells after poling.

Poling	V_{OC} [V]	FF [%]	J_{SC} [mA cm^{-2}]	PCE [%]
Control	1.138	78.9	21.3	19.1
	1.139	78.8	21.4	19.2
DCl	1.235	83.1	21.9	22.5
	1.238	83.6	21.8	22.6

Supplementary Table. 11 Summary of large-area monolithic perovskite/silicon tandems based on the spin-coating method reported in literatures. HJ: homojunction; SHJ: silicon heterojunction; TOPCon: tunnel oxide passivated contact.

PCE [%]	J_{SC} [mA cm^{-2}]	V_{OC} [V]	FF [%]	silicon	type	Area [cm^2]	Ref.
20.5	16.1	1.68	78.0	HJ	n-i-p	4	[18]
21.8	16.2	1.74	78.0	HJ	n-i-p	16	[19]
22.6	17.5	1.72	75.0	SHJ	p-i-n	57.4	[20]
23.1	16.5	1.78	81.0	HJ	n-i-p	4	[21]
27.1	19.1	1.88	75.5	SHJ	p-i-n	3.8	[22]
17.3	14.1	1.78	67.0	TOPerc	n-i-p	25	[23]
28.3	19.1	1.84	80.2	TOPCon	p-i-n	4	[24]
27.1	/	/	/	SHJ	n-i-p	24	[25]
26.4	19.0	1.91	72.6	SHJ	p-i-n	8.9	[26]
28.4	19.4	1.91	76.8	TOPCon	p-i-n	4	This work

Supplementary Table. 12 Summary of MPP stability for the state-of-the-art monolithic perovskite/silicon TSCs reported in literatures.

Structure	Environment	Encapsulation	Light condition	PCE retention	Ref.
p-i-n	In ambient air; 25–35%RH; 30 °C	no	1-sun illumination	T_{92}=100 h	27
p-i-n	In ambient air; 30–40%RH; 25 °C	no	LED with wavelengths of 470 and 940 nm	$T_{95.5}$=300 h	28
p-i-n	In N_2; 25±1 °C	no	1-sun illumination	T_{90}=100 h	29
p-i-n	In ambient air; 20%RH; 25 °C	no	1-sun illumination	T_{98}=300 h	30
p-i-n	In N_2; 25±1 °C	no	1-sun illumination	T_{118}=100 h	31
p-i-n	In N_2; 25 °C	no	White LED light source (940 nm LED light for extra compensation)	T_{98}=200 h	32
p-i-n	In ambient air; 40–70%RH; 20–35 °C	no	Xenon lamp (100 mW cm^{-2})	$T_{93.6}$=450 h	33
p-i-n	In ambient air; 25–75%RH, most often ≈60%RH; 20–35 °C	no	Xenon lamp (100 mW cm^{-2})	$T_{86.6}$=306 h	34
p-i-n	In N_2; 25 °C	no	1-sun illuminations	T_{80}=1200 h	35
p-i-n	In ambient air; 30–40%RH; 25°C	no	1-sun illuminations	T_{100}=700 h	36
p-i-n	In ambient air; 25%RH; 25°C	no	1-sun illuminations	T_{96}=527 h	37
p-i-n	In N_2; 25 °C	no	1-sun LED	T_{90}=1200 h	38

			illumination		
p-i-n	In ambient air; 40%RH; 25℃	no	1-sun illumination	$T_{90.6}$=100 h	[39]
p-i-n	In N_2; 25 °C	no	1-sun illumination	T_{95}=280 h	[40]
n-i-p	In N_2; 25 °C	no	1-sun LED illumination	T_{80}=700 h	[41]
p-i-n	In ambient air; 40–60%RH; 25–35 °C	no	1-sun xenon-lamp illumination (without a UV filter)	T_{80}=755 h	[3]
p-i-n	In ambient air; 25 °C	yes	AM 1.5G illumination	$T_{96.2}$=1068 h	[42]
p-i-n	In ambient air; 40%RH	yes	0.8-sun infrared-enhanced white LED	T_{95}=1000 h	[26]
p-i-n	In ambient air; 25%RH; 25 °C	no	1-sun illumination	T_{96}=527 h	[43]
p-i-n	In ambient air; 40–60%RH; 25–35 °C	no	1-sun xenon-lamp illumination (without a UV filter)	$T_{85.4}$=1020 h	This work

Supplementary References

1. Li Z*, et al.* Efficient charge transport in inverted perovskite solar cells via 2D/3D ferroelectric heterojunction. *Small Methods* **8**, e2400425 (2024).
2. Pica G*, et al.* Photo-ferroelectric perovskite interfaces for boosting $V_{(OC)}$ in efficient perovskite solar cells. *Nat. Commun.* **15**, 8753 (2024).
3. Li X*, et al.* Top-down dual-interface carrier management for highly efficient and stable perovskite/silicon tandem solar cells. *Nano-Micro Lett.* **17**, 141 (2025).
4. Zhao Y*, et al.* Anomalously large interface charge in polarity-switchable photovoltaic devices: an indication of mobile ions in organic–inorganic halide perovskites. *Energy Environ. Sci.* **8**, 1256–1260 (2015).
5. Li M*, et al.* Orientated crystallization of FA-based perovskite via hydrogen-bonded polymer network for efficient and stable solar cells. *Nat. Commun.* **14**, 573 (2023).
6. Kim M*, et al.* Methylammonium chloride induces intermediate phase stabilization for efficient perovskite solar cells. *Joule* **3**, 2179–2192 (2019).
7. Wang S*, et al.* Surface n-type band bending for stable inverted $CsPbI_3$ perovskite solar cells with over 20% efficiency. *Energy Environ. Sci.* **16**, 2572–2578 (2023).
8. Naqvi SDH*, et al.* Mitigating intrinsic interfacial degradation in demi-transparent perovskite solar cells for high efficiency and long-term stability. *Adv. Energy Mater.* **13**, 2302147 (2023).
9. Jia E*, et al.* Efficiency enhancement with the ferroelectric coupling effect using P(VDF-TrFE) in $CH_3NH_3PbI_3$ solar cells. *Adv. Sci.* **6**, 1900252 (2019).
10. Zhang C-C*, et al.* Polarized ferroelectric polymers for high-performance perovskite solar cells. *Adv. Mater.* **31**, 1902222 (2019).
11. Chen W*, et al.* High-polarizability organic ferroelectric materials doping for enhancing the built-in electric field of perovskite solar cells realizing efficiency over 24%. *Adv. Mater.* **34**, 2110482 (2022).
12. Xu XL*, et al.* Molecular ferroelectrics-driven high-performance perovskite solar cells. *Angew. Chem. Int. Ed.* **59**, 19974–19982 (2020).
13. Zhang H*, et al.* Highly Efficient 1D/3D Ferroelectric Perovskite Solar Cell. *Adv. Funct. Mater.* **31**, 2100205 (2021).
14. Xiao L*, et al.* In-situ organic-inorganic ferroelectric layer growth for efficient perovskite solar cells with high photovoltage. *Nano Energy* **107**, 108114 (2023).
15. Han B*, et al.* Rational design of ferroelectric 2D perovskite for improving the efficiency of flexible perovskite solar cells over 23 %. *Angew. Chem. Int. Ed.* **62**, e202217526 (2023).
16. Li Z*, et al.* Efficient charge transport in inverted perovskite solar cells via 2D/3D ferroelectric heterojunction. *Small Methods*, 2400425 (2024).
17. Pica G*, et al.* Photo-ferroelectric perovskite interfaces for boosting VOC in efficient perovskite solar cells. *Nat. Commun.* **15**, 8753 (2024).
18. Zheng J*, et al.* Large area efficient interface layer free monolithic perovskite/homo-junction-silicon tandem solar cell with over 20% efficiency. *Energy Environ. Sci.* **11**, 2432–2443 (2018).
19. Zheng J*, et al.* 21.8% Efficient monolithic perovskite/homo-junction-silicon

tandem solar cell on 16 cm^2. *ACS Energy Lett.* **3**, 2299–2300 (2018).
20. Kamino BA, *et al.* Low-temperature screen-printed metallization for the scale-up of two-terminal perovskite-silicon tandems. *ACS Appl. Energy Mater.* **2**, 3815–3821 (2019).
21. Zheng J, *et al.* Large-area 23%-efficient monolithic perovskite/homojunction-silicon tandem solar cell with enhanced UV stability using down-shifting material. *ACS Energy Lett.* **4**, 2623–2631 (2019).
22. Liu J, *et al.* 28.2%-efficient, outdoor-stable perovskite/silicon tandem solar cell. *Joule* **5**, 3169–3186 (2021).
23. Hyun JY, *et al.* Perovskite/silicon tandem solar cells with a voc of 1784 mV based on an industrially feasible 25 cm^2 TOPCon silicon cell. *ACS Appl. Energy. Mater.* **5**, 5449–5456 (2022).
24. Walter A, *et al.* Rear textured p-type high temperature passivating contacts and their implementation in perovskite/silicon tandem cells. *Energy Adv.* **2**, 1818–1822 (2023).
25. Ugur E, *et al.* Front-contact passivation through 2D/3D perovskite heterojunctions enables efficient bifacial perovskite/silicon tandem solar cells. *Matter* **6**, 2919–2934 (2023).
26. Zhu Z, *et al.* Low-temperature atomic layer deposition of hole transport layers for enhanced performance and scalability in textured perovskite/silicon tandem solar cells. *Adv. Energy Mater.* **14**, 2402365 (2024).
27. Chen B, *et al.* Blade-coated perovskites on textured silicon for 26%-efficient monolithic perovskite/silicon tandem solar cells. *Joule* **4**, 850–864 (2020).
28. Al-Ashouri A, *et al.* Monolithic perovskite/silicon tandem solar cell with >29% efficiency by enhanced hole extraction. *Science* **370**, 1300–1309 (2020).
29. Li Y, *et al.* Wide bandgap interface layer induced stabilized perovskite/silicon tandem solar cells with stability over ten thousand hours. *Adv. Energy Mater.* **11**, 2102046 (2021).
30. Li R, *et al.* $CsPbCl_3$-cluster-widened bandgap and inhibited phase segregation in a wide-bandgap perovskite and its application to NiOx-based perovskite/silicon tandem solar cells. *Adv. Mater.* **34**, 2201451 (2022).
31. Ren N, *et al.* 50 °C low-temperature ALD SnO_2 driven by H_2O_2 for efficient perovskite and perovskite/silicon tandem solar cells. *Appl. Phys. Lett.* **121**, 033502 (2022).
32. Ji SG, *et al.* Stable pure-iodide wide-band-gap perovskites for efficient Si tandem cells via kinetically controlled phase evolution. *Joule* **6**, 2390–2405 (2022).
33. Wang X, *et al.* Long-chain anionic surfactants enabling stable perovskite/silicon tandems with greatly suppressed stress corrosion. *Nat. Commun.* **14**, 2166 (2023).
34. Li X, *et al.* Surface reconstruction for efficient and stable monolithic perovskite/silicon tandem solar cells with greatly suppressed residual strain. *Adv. Mater.* **35**, e2211962 (2023).
35. Liu J, *et al.* Perovskite/silicon tandem solar cells with bilayer interface

passivation. *Nature* **635**, 596–603 (2024).
36. Liu J*, et al.* Textured perovskite/silicon tandem solar cells achieving over 30% efficiency promoted by 4-fluorobenzylamine hydroiodide. *Nano-Micro Lett.* **16**, 189 (2024).
37. Pei F*, et al.* A binary 2D perovskite passivation for efficient and stable perovskite/silicon tandem solar cells. *Nat. Commun.* **15**, 7024 (2024).
38. Sun Y*, et al.* Ionic liquid modified polymer intermediate layer for improved charge extraction toward efficient and stable perovskite/silicon tandem solar cells. *Small* **20**, 2308553 (2024).
39. Wang X*, et al.* Ultrathin (~30 μm) flexible monolithic perovskite/silicon tandem solar cell. *Sci. Bull.* **69**, 1887–1894 (2024).
40. Yang T*, et al.* Efficient and stable perovskite/silicon tandem solar cells modulated with triple-functional passivator. *Adv. Energy Mater.* **14**, 2303149 (2023).
41. Ding Z*, et al.* Highly passivated TOPCon bottom cells for perovskite/silicon tandem solar cells. *Nat. Commun.* **15**, 8453 (2024).
42. Ye T*, et al.* Molecular bridge in wide-bandgap perovskites for efficient and stable perovskite/silicon tandem solar cells. *Adv. Funct. Mater.* **35**, 2419391 (2025).
43. Pei F*, et al.* A binary 2D perovskite passivation for efficient and stable perovskite/silicon tandem solar cells. *Nat. Commun.* **15**, 7024 (2024).
