# Supplementary material for: Minimizing interfacial energy losses via multifunctional cage-like diammonium molecules for efficient perovskite/silicon tandem solar cells
Source: Nat Commun. 2025 Sep 30;16:8692. doi: 10.1038/s41467-025-63720-8 (PMC12484657; doi:10.1038/s41467-025-63720-8)
Supplement: Supplementary file 2 — Reporting Summary [file 41467_2025_63720_MOESM2_ESM.pdf]

nature portfolio

Corresponding author(s): Zhiqin Ying, Xi Yang, Jichun Ye

Solar Cells Reporting Summary

Nature Portfolio wishes to improve the reproducibility of the work that we publish. This form is intended for publication with all accepted papers reporting the characterization of photovoltaic devices and provides structure for consistency and transparency in reporting. Some list items might not apply to an individual manuscript, but all fields must be completed for clarity.

For further information on Nature Research policies, including our data availability policy, see Authors & Referees.

Experimental design

Please check the following details are reported in the manuscript, and provide a brief description or explanation where applicable.

1. Dimensions

Area of the tested solar cells

☒ Yes ☐ No

0.1 cm2 for single junction and 1.0 cm2 for tandems (Methods)

Explain why this information is not reported/not relevant.

Method used to determine the device area

☒ Yes ☐ No

The device areas were defined by masks with different aperture areas (Methods)

Explain why this information is not reported/not relevant.

2. Current-voltage characterization

Current density-voltage (J-V) plots in both forward and backward direction

☒ Yes ☐ No

J-V curves were obtained from scans in both forward and backward direction. The J-V curves can be found in Fig. 4 and supplementary information.

Voltage scan conditions

☒ Yes ☐ No

All J–V measurements were conducted at a scan rate of 10 mV/s with the delay time of 10 ms

Explain why this information is not reported/not relevant.

Test environment

☒ Yes ☐ No

Our devices were characterized at room temperature (ca. 25 Celsius degree) in air

Explain why this information is not reported/not relevant.

Protocol for preconditioning of the device before its characterization

☒ Yes ☐ No

No preconditioning protocol was used in the characterization. The information is stated in “device characterization” part in “Methods”

Explain why this information is not reported/not relevant.

Stability of the J-V characteristic

☒ Yes ☐ No

Stabilized photocurrent output by holding the voltage at the maximum power point (Supplementary Figs. 36, 53, 56)

Explain why this information is not reported/not relevant.

3. Hysteresis or any other unusual behaviour

Description of the unusual behaviour observed during the characterization

☒ Yes ☐ No

No J-V hysteresis or any other unusual behaviour was observed and the related comments were mentioned in manuscript.

Explain why this information is not reported/not relevant.

Related experimental data

☒ Yes ☐ No

We show the J-V curves under different directions in Fig. 4 and supplementary information.

Explain why this information is not reported/not relevant.

4. Efficiency

External quantum efficiency (EQE) or incident photons to current efficiency (IPCE)

☒ Yes ☐ No

The EQE spectrum can be found in supplementary Figs. 37, 40, 44, 45 and 54.

Explain why this information is not reported/not relevant.

A comparison between the integrated response under the standard reference spectrum and the response measure under the simulator

☒ Yes ☐ No

The integrated JSC from EQE spectrum are agreed well (less than 5% mismatch) with the J-V measurements. The comparison is represented in supplementary Figs. 37, 40, 44, 45 and 54.

Explain why this information is not reported/not relevant.

For tandem solar cells, the bias illumination and bias voltage used for each subcell

☒ Yes ☐ No

"Device characterization" part in "Methods"

Explain why this information is not reported/not relevant.

5. Calibration

Light source and reference cell or sensor used for the characterization

☒ Yes ☐ No

A solar simulator (EMS–35AAA, Ushio Spax Inc.) based on the Ushio Xe short arc lamp 500 was used to simulate sunlight irradiation of 1 sun (AM1.5G; 100 mW cm-2). The solar simulator illumination intensity was calibrated using a KG5 reference Si-cell (Enlitech). The information can be found in the "Device characterization" part in "Methods"

Explain why this information is not reported/not relevant.

Confirmation that the reference cell was calibrated and certified

☒ Yes ☐ No

The information can be found in the "Device characterization" part in "Methods"

Explain why this information is not reported/not relevant.

Calculation of spectral mismatch between the reference cell and the devices under test

☒ Yes ☐ No

Spectral mismatch factor of 1 was used for all J-V measurements

Explain why this information is not reported/not relevant.

6. Mask/aperture

Size of the mask/aperture used during testing

☒ Yes ☐ No

Single junction solar cells:0.1 cm2, 1.21 cm2; Tandem solar cells:1.0 cm2

Explain why this information is not reported/not relevant.

Variation of the measured short-circuit current density with the mask/aperture area

☒ Yes ☐ No

J-V results in main text are reported with illumination mask. We note, the unmasked current is typically ~5% higher.

Explain why this information is not reported/not relevant.

7. Performance certification

Identity of the independent certification laboratory that confirmed the photovoltaic performance

☒ Yes ☐ No

We sent our device to Ningbo New Materials Testing and Evaluation Center (NBMTEC) for j-V characterization according to their standard protocol (IEC60891: 2021).The information can be found in Supplementary Fig.55.

Explain why this information is not reported/not relevant.

A copy of any certificate(s)

☒ Yes ☐ No

NBMTEC provided j-V results according to their standard protocol IEC60891: 2021). The information can be found in Supplementary Fig. 55.

Explain why this information is not reported/not relevant.

8. Statistics

Number of solar cells tested

☒ Yes ☐ No

22 devices are provided for statistical analysis of the photovoltaic parameters

Explain why this information is not reported/not relevant.

Statistical analysis of the device performance

☒ Yes ☐ No

Supplementary Fig. 39

Explain why this information is not reported/not relevant.

9. Long-term stability analysis

Type of analysis, bias conditions and environmental conditions

☒ Yes ☐ No

The unencapsulated tandems were aged using a xenon lamp with 100 mW cm-2 irradiance at the regular ambient conditions (40-60%RH, 25-35?) without preconditioning. The information of long-term stability test is presented in the manuscript and in the "Methods" section.

Explain why this information is not reported/not relevant.
